# Safety and efficacy of different JAK inhibitors in the treatment of inflammatory bowel disease: a network meta-analysis

**DOI:** 10.3389/fphar.2025.1699928

**Published:** 2026-01-27

**Authors:** Haidong Wu, Yongci Zhou, Xuyong Chen, Liudan Wang, Yifan Guo, Xiaxia Du, Xinpu Miao

**Affiliations:** 1 Department of Gastroenterology, Hainan General Hospital, Hainan Affiliated Hospital of Hainan Medical University, Haikou, Hainan, China; 2 Department of Otorhinolaryngology Head and Neck Surgery, Hainan General Hospital, Hainan Affiliated Hospital of Hainan Medical University, Haikou, Hainan, China; 3 Department of the Center of Gerontology, Hainan General Hospital, Hainan Affiliated Hospital of Hainan Medical University, Haikou, Hainan, China

**Keywords:** efficacy, inflammatory bowel disease, JAK inhibitor, network meta-analysis, safety

## Abstract

Targeting the Janus kinase (JAK) signaling pathway is a new way to treat inflammatory bowel disease (IBD). This network meta-analysis aimed to compare the efficacy and safety of various JAK inhibitors, including brepocitinib, filgotinib, ivarmacitinib, peficitinib, ritlecitinib, tofacitinib, and upadacitinib, in patients with IBD. The analysis included patients with both Crohn’s disease (CD) and ulcerative colitis (UC). We systematically searched PubMed, Embase, the Cochrane Library, and the Web of Science for randomized controlled trials evaluating JAK inhibitors in patients with CD or UC up to 4 May 2024. A pooled analysis of UC and CD was performed. The primary outcome was clinical remission. Secondary outcomes included clinical response, endoscopic remission, endoscopic response, endoscopic improvement, adverse events (AEs), serious adverse events (SAEs), AEs leading to treatment discontinuation, and infections. Ranking was assessed using the surface under the cumulative ranking curve (SUCRA) probabilities. Ritlecitinib exhibited the highest SUCRA probabilities for clinical remission (88.7%), clinical response (86.0%), and endoscopic improvement (92.1%). Upadacitinib exhibited superiority in endoscopic remission (85.6%) and response (99.5%), demonstrating moderate efficacy in clinical response (82.2%). A safety analysis revealed comparable AE rates for most agents compared to placebo, except for upadacitinib and brepocitinib. Tofacitinib showed the lowest risk of SAEs (72.3%). Upadacitinib had the lowest discontinuation rate (81.3%). Ivarmacitinib demonstrated optimal infection safety (80.3%). Upadacitinib has the best efficacy-safety profile for IBD, while ritlecitinib’s superior efficacy is offset by higher safety risks. Long-term studies are needed to confirm these results.

**Systematic Review Registration:**

https://www.crd.york.ac.uk/PROSPERO/view/CRD42024595343, Identifier CRD42024595343.

## Introduction

The term “inflammatory bowel disease” (IBD) encompasses both ulcerative colitis (UC) and Crohn’s disease (CD). These chronic, nonspecific intestinal inflammatory diseases have an unclear underlying cause (Le Berre et al., 2023; Dolinger et al., 2024). IBD is a significant public health concern on a global scale. The rising prevalence of IBD places a significant financial burden on society, families, and individuals alike (Ng et al., 2017; Zhang et al., 2024; Bisgaard et al., 2022). Therefore, IBD treatment has become a key area of study. Previously, drug treatments for IBD primarily included 5-aminosalicylic acid, glucocorticoids, immunosuppressants, and biologics (Vetter and Neurath, 2017). 5-aminosalicylic acid is frequently utilized for the induction and remission treatment of mild IBDs. Glucocorticoids are the mainstay of treatment for moderate to severe IBD. Immunosuppressants and biologics are often used to maintain remission in patients with moderate to severe IBD. However, these drugs have several drawbacks, including poor efficacy, a high recurrence rate, and significant adverse effects, particularly for biologics, which are also expensive (Salas et al., 2020; Hirten and Sands, 2021). As knowledge of the physiological and pathological mechanisms of IBD expands, Janus kinase (JAK) drugs have introduced new possibilities for treating the disease. JAK inhibitors have been approved in multiple countries for treating IBD, marking the first time a small-molecule drug has been approved for this indication (Herrera-deGuise et al., 2023). JAK comprises four key components: JAK1, JAK2, JAK3, and tyrosine kinase 2 (TYK2). JAKs are a group of tyrosine protein kinases that mediate inflammatory responses (De Vries et al., 2017). When cytokines bind to cell-surface receptors, they activate JAKs. The JAKs then phosphorylate the signal transducer and activator of transcription (STAT) protein. This phosphorylation triggers the release of the STAT protein from the receptor. The released STAT subsequently dimerizes and translocates to the cell nucleus, where it initiates the transcription of pro-inflammatory mediators (Salas et al., 2020; O'Shea et al., 2013; Roskoski, 2016; Schwartz et al., 2017; McLornan et al., 2021). A substantial body of evidence supports the efficacy of JAK inhibitors in treating IBD (Bonelli et al., 2024). JAK inhibitors have several advantages, including a lack of antigenicity, oral administration, and a short half-life. However, due to the complex disease mechanisms, uncertain efficacy, and safety concerns, previously approved JAK inhibitors, such as tofacitinib and filgotinib, were only used to treat UC (Mannucci et al., 2022; Steenholdt et al., 2023). The introduction of upadacitinib has changed this landscape. Upadacitinib has high selectivity for JAK1 inhibition. This makes it the only JAK inhibitor approved for both CD and UC. Meanwhile, additional JAK inhibitors (e.g., ABT-494, brepocitinib, ivarmacitinib, peficitinib, and ritlecitinib) are still undergoing clinical trials for the treatment of IBD (Nash et al., 2021; Olivera et al., 2017; Núñez et al., 2023). Although many JAK inhibitors are available, head-to-head comparisons are currently lacking. There is also a lack of consensus on which JAK inhibitor is more effective for patients with IBD or which has fewer adverse reactions. For this reason, the current study was conducted to clarify the relative efficacy and safety profiles of different JAK inhibitors for treating IBD through a network meta-analysis (NMA). The goal is to provide IBD patients with new treatment options.

## Methods and data

### Literature search

A comprehensive search of the Web of Science, Cochrane, PubMed, and Embase databases was conducted to identify randomized controlled trials (RCTs) examining the effects of JAK inhibitors on IBD (including CD and UC) up to 4 May 2024. The search covered the following terms, combining free words and subject words: “Inflammatory Bowel Diseases”, “Janus Kinase Inhibitors.” The detailed search strategy is provided in Supplementary Material 1.

### Eligibility criteria

Inclusion criteria: (1) Subjects who met the diagnostic criteria for IBD. (2) The intervention measures were JAK inhibitors; placebo or prednisolone was used in the control group. (3) RCTs. (4) The primary outcome was clinical remission (UC: a total Mayo score of ≤2, with no individual subscore >1; CD: a CDAI score of <150). Secondary outcomes included clinical response (UC: a reduction of at least 3 points and at least 30% in the overall Mayo score, accompanied by a reduction in the rectal bleeding subscore of at least 1 point or an absolute rectal bleeding subscore of 0 or 1; CD: a decrease in the CDAI score of ≥100 points from baseline), endoscopic remission (UC: a Mayo endoscopic subscore of 0; CD: a simple endoscopic score for Crohn’s disease [SES-CD] of ≤4, a decrease of ≥2 points from baseline with no subscore >1), endoscopic response (UC: a decrease from baseline in the endoscopy subscore by at least 1; CD: a decrease in the SES-CD of more than 50% from baseline), endoscopic improvement (UC: a Mayo endoscopic subscore of ≤1 point), adverse events (AEs), serious adverse events (SAEs), AEs leading to treatment discontinuation, and infections.

Exclusion criteria: Full text unavailable, duplicate literature, studies combining other organic diseases, studies unrelated to JAK inhibitors and IBD, reviews, conference abstracts, case reports, and animal studies.

### Data extraction

Two authors conducted a comprehensive literature review in accordance with predefined inclusion and exclusion criteria. Any discrepancies were resolved through discussion or by seeking the advice of a third party. They extracted the following information: publication year, first author, registration number, sample size, sex, country, age, intervention, and outcome indicators.

### Quality evaluation

The latest recommendations from the Cochrane Risk of Bias Assessment Tool 2.0 (ROB 2.0) (Sterne et al., 2019) were adopted to evaluate the ROB. The tool has five main components: bias due to randomization, bias due to selective reporting of outcomes, bias due to intervention deviation, bias due to outcome measurement, and bias due to missing data on outcomes. The criteria for evaluating the quality of the literature were as follows: low ROB, partially noteworthy, and high ROB. Two researchers reviewed the findings of the study. If discrepancies arose, the researchers discussed them to reach a resolution or consulted a third party.

### Data analysis

A Bayesian NMA was performed using the prior fuzzy random effects model implemented in R4.3.2 software (R Foundation for Statistical Computing) on multiple groups of studies. To obtain the most precise combined estimates and probabilities for each treatment regimen, this NMA employed the Markov chain Monte Carlo method (Jansen et al., 2008). Model convergence was evaluated using trajectory plots and Brooks-Gelman-Rubin plots. The odds ratio (OR) and its 95% confidence interval (CI) were used to express the results of the continuity analysis. The probability of an intervention being optimal was determined by computing the surface under the cumulative ranking curve (SUCRA). Higher SUCRA values indicate superior efficacy or safety. Funnel and network diagrams were created using STATA 15.0 and a pass-through macro command. In a network diagram, each drug is represented by a circle, and existing comparisons are indicated by edges. The size of each circle is proportional to the number of included patients. Cumulative probability was plotted using the ggplot2 package.

## Results

### Data screening and results

**FIGURE 1:**
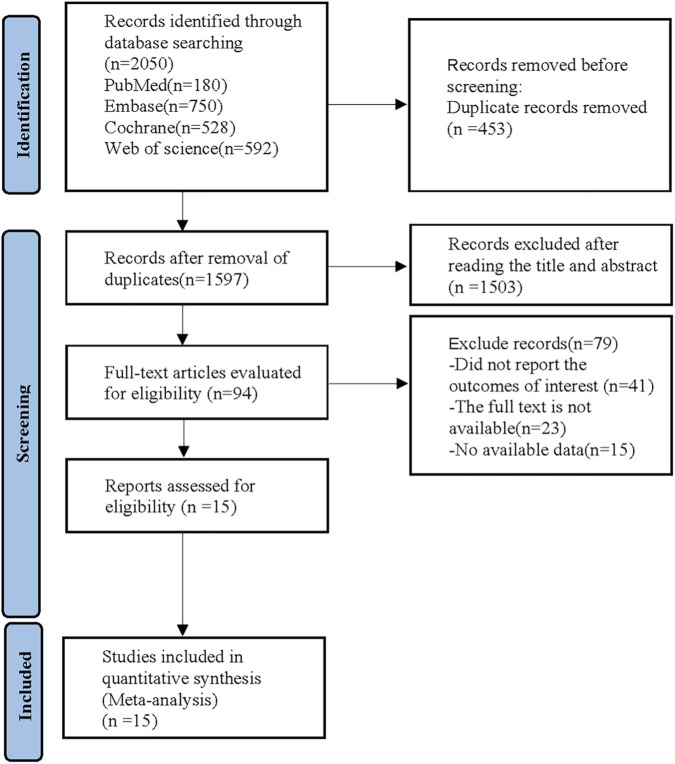
Literature screening process.

The initial database search yielded 2,050 articles. First, 453 duplicates were removed. Then, 1,503 articles were excluded by examining the titles and abstracts. Subsequently, 79 articles were removed by reading the full text. Ultimately, 15 articles were included in this NMA (Chen et al., 2022; Danese et al., 2022; Feagan et al., 2021; Loftus et al., 2023; Panés et al., 2017; Reinisch et al., 2024; Sandborn et al., 2023; Sandborn et al., 2020; Sandborn et al., 2012; Sandborn et al., 2014; Sandborn et al., 2017; Sands et al., 2018; Singh et al., 2024a; Singh et al., 2024b; Vermeire et al., 2017) (Figure 1).

### Basic characteristics of literature and deviation risk assessment

**FIGURE 2:**
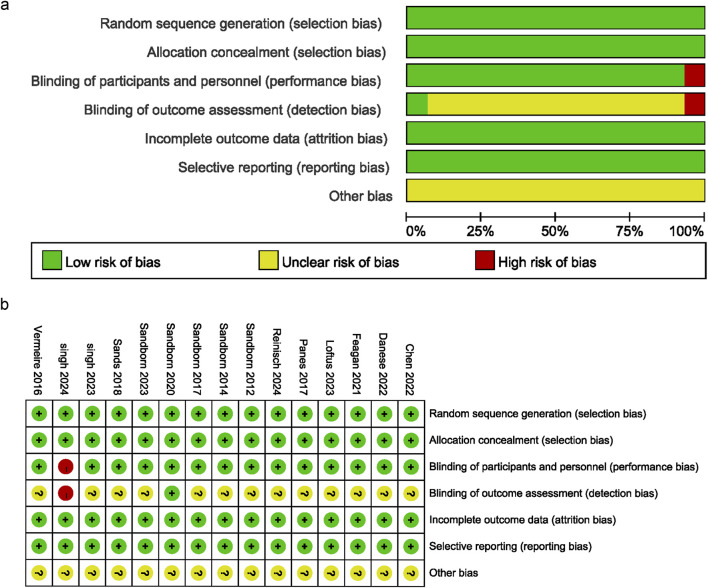
Risk of bias graph. **(a)** Bias risk assessment chart, **(b)** Summary of bias risk across studies.

The analysis included 15 articles with data from 6,466 patients with IBD. The JAK inhibitor drugs examined were brepocitinib, filgotinib, ivarmacitinib, peficitinib, ritlecitinib, tofacitinib, and upadacitinib. Their doses ranged from 0.5 to 200 mg. Table 1 provides a detailed overview of the literature’s specific characteristics. All of the studies included in the current analysis provided clear and detailed explanations of the blinding method. The main risk was the potential for deviations from the expected intervention. Figures 2a,b present a detailed deviation risk assessment of the included studies.

**TABLE 1:** Characteristics of the included studies.

Study	Year	Registration number	Country	Sample size	Gender (M/F)	Mean age (years)	Intervention	Outcome
Sandborn	2012	NCT00787202	America	Tofacitinib: 146 Placebo: 48	106/88	Tofacitinib:42.5 Placebo:42.5	Tofacitinib:Orally; BID; 8 weeks	F1; F2; F3; F4; F6; F7; F8; F9
Sandborn	2014	NCT00615199	America	Tofacitinib: 105 Placebo: 34	69/70	Tofacitinib: 37.8 Placebo: 35.7	Tofacitinib:Orally; BID; 4 weeks	F1; F2; F6; F7; F8
Vermeire	2016	NCT02048618	Belgium	Filgotinib: 130 Placebo: 44	77/97	Filgotinib: 37.4 Placebo: 35.1	Filgotinib:200 mg; Orally; QD; 10 weeks	F1; F2; F3; F4; F6; F7; F8; F9
Panés	2017	NCT01393626, NCT01393899	Spain	Tofacitinib: 188 Placebo: 91	133/146	Tofacitinib:39.9 Placebo:37.2	Tofacitinib:Orally; BID; 8 weeks	F2; F6; F7; F8; F9
Sandborn	2017	NCT01465763, NCT01458951, NCT01458574	America	Tofacitinib: 476 Placebo: 122	354/244	Tofacitinib:41.3 Placebo:41.8	Tofacitinib:10 mg; Orally; BID; 8 weeks	F1; F2; F3; F5; F6; F7; F8; F9
				Tofacitinib: 429 Placebo: 112	314/227	Tofacitinib:41.1 Placebo:40.4	Tofacitinib:10 mg; Orally; BID; 8 weeks	F1; F2; F3; F5; F6; F7; F8; F9
Sands	2018	NCT01959282	America	Peficitinib: 176 Placebo: 43	130/89	Peficitinib:41 Placebo:39	Peficitinib:Orally; 8weeks	F1; F2; F3; F5; F6; F7; F8; F9
Sandborn	2020	NCT02365649	America	Upadacitinib: 183 Placebo: 37	95/125	Upadacitinib: 40.3 Placebo: 40	Upadacitinib:Orally; 16 weeks	F1; F2; F3; F4; F6; F7; F8; F9
Feagan	2021	NCT02914522	Canada	Filgotinib: 522 Placebo: 137	367/292	Filgotinib:42 Placebo:41	Filgotinib:Orally; QD; 11 weeks;	F1; F2; F3; F5; F6; F7; F8; F9
				Filgotinib: 547 Placebo: 142	420/269	Filgotinib:43 Placebo:44	Filgotinib:Orally; QD; 11 weeks;	F1; F2; F3; F5; F6; F7; F8; F9
Chen	2022	NCT03675477	China	Ivarmacitinib:123 Placebo: 41	100/64	Ivarmacitinib:39.8 Placebo:42.7	Ivarmacitinib:Orally; 8 weeks;	F1; F2; F3; F5; F6; F7; F8; F9
Danese	2022	NCT02819635, NCT03653026	Italy	Upadacitinib: 319 Placebo: 154	295/178	Upadacitinib:43 Placebo:44.5	Upadacitinib:45 mg; Orally; QD; 8weeks	F1; F2; F3; F5; F6; F7; F8; F9
				Upadacitinib: 341 Placebo: 174	321/194	Upadacitinib:40 Placebo:42	Upadacitinib:45 mg; Orally; QD; 8weeks	F1; F2; F3; F5; F6; F7; F8; F9
Loftus	2023	NCT03345849, NCT03345836, NCT03345823	America	Upadacitinib: 350 Placebo: 176	283/243	Upadacitinib: 39.7 Placebo: 39.3	Upadacitinib: 45 mg; Orally; QD; 12 weeks	F1; F2; F3; F4; F6; F7; F8; F9
				Upadacitinib: 324 Placebo: 171	265/230	Upadacitinib: 38.4 Placebo: 37.5	Upadacitinib: 45 mg; Orally; QD; 12 weeks	F1; F2; F3; F4; F6; F7; F8; F9
Sandborn	2023	NCT02958865	America	Ritlecitinib: 150 Placebo: 25	104/71	Ritlecitinib:39.6 Placebo:42.8	Ritlecitinib:Orally; QD; 8 weeks	F1; F2; F3; F5; F6; F7; F8; F9
				Brepocitinib: 142 Placebo: 25	92/75	Brepocitinib:40.7 Placebo:42.8	Brepocitinib:Orally; QD; 8 weeks	F1; F2; F3; F5; F6; F7; F8; F9
Singh	2023	ISRCTN42182437	India	Tofacitinib: 53 Placebo: 51	59/45	Tofacitinib:37 Placebo:38	Tofacitinib:10 mg; Orally; TID; 7days	F6; F7; F8; F9
Reinisch	2024	NCT03077412	Austria	Filgotinib: 42 Placebo: 15	34/23	Filgotinib:40.2 Placebo:39	Filgotinib:Orally; QD; 24 weeks	F6; F7; F8; F9
Singh	2024	CTRI/2021/10/037641	India	Tofacitinib: 43 Prednisolone: 35	42/36	Tofacitinib:37.6 Prednisolone:39.2	Tofacitinib:10 mg; Orally; BID; 8weeks	F1; F2; F3; F5; F6; F7; F9

M/F: Male/Female.

F1: Clinical response.

F2: Clinical remission.

F3: Endoscopic remission.

F4: Endoscopic response.

F5: Endoscopic improvement.

F6: Adverse events.

F7: Serious adverse events.

F8: Adverse events leading to treatment discontinuation.

F9: Infections.

### NMA results

#### Efficacy

##### Clinical remission

**FIGURE 3:**
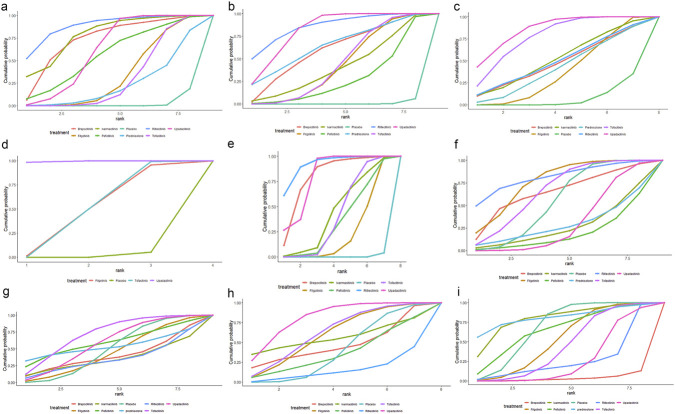
**(a)** Line chart of clinical remission; **(b)** Line chart of clinical response; **(c)** Line chart of endoscopic remission; **(d)** Line chart of endoscopic response; **(e)** Line chart of endoscopic improvement; **(f)** Line chart of adverse events; **(g)** Line chart of serious adverse events; **(h)** Line chart of adverse events leading to treatment discontinuation; **(i)** Line chart of infections.

Fourteen articles reported clinical remission, as illustrated in Supplementary Figure S1a. Since the closed loops in the network originated solely from within-study comparisons, inconsistency testing was unnecessary. Compared to placebo, ritlecitinib [OR = 11.1, 95% CI (1.84, 373.)], brepocitinib [OR = 7.86, 95% CI (1.28, 262.)], ivarmacitinib [OR = 7.05, 95% CI (1.86, 51.9)], upadacitinib [OR = 3.46, 95% CI (2.74, 4.40)], peficitinib [OR = 3.41, 95% CI (1.10, 15.6)], filgotinib [OR = 2.10, 95% CI (1.45, 3.13)], and tofacitinib [OR = 1.99, 95%CI (1.49, 2.70)] were associated with significantly higher rates of clinical remission in patients with IBD (Supplementary Figure S1b), and upadacitinib was superior to filgotinib and tofacitinib (Table 2). Based on SUCRA values, ritlecitinib had the highest value (88.7%), followed by ivarmacitinib (78.8%), brepocitinib (73.7%), upadacitinib (61.7%), peficitinib (56.8%), filgotinib (33.9%), tofacitinib (30.5%), and placebo (2.4%) (Figure 3a; Table 3).

**TABLE 2:** League table of clinical remission.

OR 95%CI								
Brepocitinib	​	​	​	​	​	​	​	​
3.74 (0.58, 127.76)	Filgotinib	​	​	​	​	​	​	​
1.12 (0.08, 47.17)	0.3 (0.04, 1.2)	Ivarmacitinib	​	​	​	​	​	​
2.33 (0.21, 89.26)	0.62 (0.13, 2.06)	2.08 (0.28, 20.05)	Peficitinib	​	​	​	​	​
7.86 (1.28, 262.26)*	2.1 (1.45, 3.13)*	7.05 (1.86, 51.86)*	3.41 (1.1, 15.6)*	Placebo	​	​	​	​
5 (0.6, 185.77)	1.3 (0.45, 3.75)	4.43 (0.82, 39.56)	2.14 (0.46, 12.57)	0.61 (0.23, 1.64)	Prednisolone	​	​	​
0.71 (0.4, 1.24)	0.19 (0.01, 1.2)	0.63 (0.02, 9.29)	0.3 (0.01, 3.29)	0.09 (0, 0.54)*	0.14 (0, 1.15)	Ritlecitinib	​	​
3.96 (0.63, 132.04)	1.06 (0.65, 1.73)	3.55 (0.9, 26.41)	1.71 (0.53, 8)	0.5 (0.37, 0.67)*	0.82 (0.32, 2.05)	5.6 (0.9, 187.79)	Tofacitinib	​
2.28 (0.36, 76.5)	0.61 (0.39, 0.97)*	2.04 (0.53, 15.07)	0.99 (0.31, 4.59)	0.29 (0.23, 0.37)*	0.47 (0.17, 1.27)	3.22 (0.52, 109.3)	0.58 (0.39, 0.84)*	Upadacitinib

*Means P < 0.05.

**TABLE 3:** SUCRA ranking for overall inflammatory bowel disease analysis.

Treatment	Clinical reminssion (%)	Clinical response (%)	Endoscopic remission (%)	Endoscopic response (%)	Endoscopic improvement (%)	Adverse events (%)	Serious adverse events (%)	Adverse events leading to treatment discontinuation (%)	Infections (%)
Brepocitinib	73.7	60	48	-	79.9	65	40.1	46.8	3.5
Filgotinib	33.9	43.1	37.4	49	24.2	76.3	44.9	60.3	52.8
Ivarmacitinib	78.8	41.2	51.9	-	44.8	26.8	35.3	56.2	80.3
Peficitinib	56.8	27.7	-	-	36	18.9	60	37.1	65.5
Prednisolone	23.4	64.7	42.2	-	-	29.1	53.8	-	81.4
Ritlecitinib	88.7	86	49.9	-	92.1	81.5	35.5	15.4	26.7
Tofacitinib	30.5	44.4	77.7	49.7	42.2	66.7	72.3	62.8	44.9
Upadacitinib	61.7	82.2	85.6	99.5	80.2	30.9	59	81.3	26.6
Placebo	2.4	0.8	7.4	1.7	0.6	54.7	49.1	40	68.4

##### Clinical response

As illustrated in Supplementary Figure S2a, 12 articles reported clinical response. Given that all closed loops originated from within-study comparisons, assessment of inconsistency was not required. Compared to placebo, ritlecitinib [OR = 4.80, 95% CI (1.89, 14.)], upadacitinib [OR = 4.10, 95% CI (3.38, 5.0)], brepocitinib [OR = 3.28, 95% CI (1.29, 9.56)], tofacitinib [OR = 2.51, 95% CI (1.94, 3.27)], filgotinib [OR = 2.47, 95% CI (1.90, 3.23)], and ivarmacitinib [OR = 2.33, 95% CI (1.09, 5.24)] were associated with significantly higher rates of clinical response in patients with IBD (Supplementary Figure S2b), and upadacitinib was superior to tofacitinib, peficitinib and filgotinib (Table 4). Based on SUCRA values, ritlecitinib had the highest value (86.0%), followed by upadacitinib (82.2%), brepocitinib (60.0%), tofacitinib (44.4%), filgotinib (43.1%), ivarmacitinib (41.2%), peficitinib (27.7%), and placebo (0.80%) (Figure 3b; Table 3).

**TABLE 4:** League table for clinical response.

OR 95%CI								
Brepocitinib	​	​	​	​	​	​	​	​
1.33 (0.5, 3.99)	Filgotinib	​	​	​	​	​	​	​
1.42 (0.41, 5.23)	1.06 (0.45, 2.39)	Ivarmacitinib	​	​	​	​	​	​
1.74 (0.54, 6.12)	1.3 (0.62, 2.69)	1.23 (0.44, 3.52)	Peficitinib	​	​	​	​	​
3.28 (1.29, 9.56)*	2.47 (1.9, 3.23)*	2.33 (1.09, 5.24)*	1.9 (0.96, 3.81)	Placebo	​	​	​	​
0.97 (0.24, 4.16)	0.73 (0.25, 2.04)	0.69 (0.19, 2.49)	0.56 (0.16, 1.89)	0.29 (0.1, 0.8)*	Prednisolone	​	​	​
0.68 (0.43, 1.09)	0.52 (0.17, 1.36)	0.48 (0.13, 1.68)	0.39 (0.11, 1.27)	0.21 (0.07, 0.53)*	0.7 (0.17, 2.84)	Ritlecitinib	​	​
1.3 (0.5, 3.92)	0.98 (0.68, 1.42)	0.93 (0.41, 2.18)	0.75 (0.36, 1.59)	0.4 (0.31, 0.51)*	1.35 (0.51, 3.68)	1.91 (0.73, 5.73)	Tofacitinib	​
0.8 (0.31, 2.37)	0.6 (0.43, 0.84)*	0.57 (0.26, 1.31)	0.46 (0.23, 0.95)*	0.24 (0.2, 0.3)*	0.83 (0.3, 2.37)	1.17 (0.45, 3.45)	0.61 (0.44, 0.85)*	Upadacitinib

*Means P < 0.05.

##### Endoscopic remission

Ten articles reported endoscopic remission, as illustrated in Supplementary Figure S3a. Closed loops in the network originated solely from within-study comparisons, rendering inconsistency testing unnecessary. Compared with placebo, upadacitinib [OR = 8.12, 95% CI (5.35, 13.1)], tofacitinib [OR = 6.34, 95% CI (2.75, 18.9)], and filgotinib [OR = 2.17, 95% CI (1.18, 4.41)] were associated with significantly higher rates of endoscopic remission in IBD patients (Supplementary Figure S3b), and upadacitinib was superior to filgotinib (Table 5). Based on SUCRA values, the highest value was observed for upadacitinib (85.6%), followed by tofacitinib (77.7%), ivarmacitinib (51.9%), ritlecitinib (49.9%), brepocitinib (48.0%), filgotinib (37.4%), and placebo (7.40%) (Figure 3c; Table 3).

**TABLE 5:** League table for endoscopic remission.

OR 95%CI							
Brepocitinib	​	​	​	​	​	​	​
1.27 (0.17, 39.95)	Filgotinib	​	​	​	​	​	​
0.88 (0.06, 33.12)	0.69 (0.09, 3.34)	Ivarmacitinib	​	​	​	​	​
2.74 (0.42, 83.95)	2.17 (1.18, 4.41)*	3.14 (0.78, 23.01)	Placebo	​	​	​	​
1.12 (0.09, 44.77)	0.85 (0.15, 5.17)	1.26 (0.14, 15.94)	0.39 (0.08, 2.08)	Prednisolone	​	​	​
0.96 (0.4, 2.28)	0.76 (0.02, 5.61)	1.09 (0.03, 16.76)	0.35 (0.01, 2.23)	0.85 (0.02, 10.75)	Ritlecitinib	​	​
0.43 (0.05, 14.34)	0.34 (0.1, 1.02)	0.49 (0.08, 4.25)	0.16 (0.05, 0.36)*	0.4 (0.1, 1.38)	0.45 (0.05, 14.48)	Tofacitinib	​
0.34 (0.05, 10.56)	0.27 (0.12, 0.61)*	0.39 (0.09, 2.94)	0.12 (0.08, 0.19)*	0.32 (0.06, 1.67)	0.35 (0.05, 10.66)	0.78 (0.3, 2.47)	Upadacitinib

*Means P < 0.05.

##### Endoscopic response

As illustrated in Supplementary Figure S4a, four articles reported endoscopic response. Since the network diagram showed no closed loop, the local inconsistency testing was not conducted. Upadacitinib [OR = 7.78, 95% CI (5.25, 11.9)] and tofacitinib [OR = 2.21, 95% CI (1.14, 4.34)] led to a significantly better endoscopic response compared to placebo in patients with IBD (Supplementary Figure S4b). Upadacitinib was also superior to tofacitinib and filgotinib (Table 6). Based on SUCRA values, upadacitinib had the highest value (99.5%), followed by tofacitinib (49.7%), filgotinib (49.0%), and placebo (1.7%) (Figure 3d; Table 3).

**TABLE 6:** League table for endoscopic response.

OR 95%CI			
Filgotinib	​	​	​
2.21 (0.89, 6.34)	Placebo	​	​
1 (0.32, 3.46)	0.45 (0.23, 0.88)*	Tofacitinib	​
0.28 (0.1, 0.87)*	0.13 (0.08, 0.19)*	0.28 (0.13, 0.62)*	Upadacitinib

*Means P < 0.05.

##### Endoscopic improvement

This outcome was only observed in UC. Seven articles reported endoscopic improvement, as shown in Supplementary Figure S5a. Since all closed loops in the network were formed by within-study comparisons, inconsistency was not evaluated. Compared with placebo, ritlecitinib [OR = 16, 95% CI (2.69, 441.)], brepocitinib [OR = 12.4, 95% CI (2.08, 347.)], upadacitinib [OR = 8.44, 95% CI (5.54, 13.3)], ivarmacitinib [OR = 2.72, 95% CI (1.11, 7.77)], tofacitinib [OR = 2.46, 95% CI (1.73, 3.57)], and filgotinib [OR = 1.81, 95% CI (1.26, 2.66)] resulted in significantly better endoscopic improvement in patients with IBD (Supplementary Figure S5b). Upadacitinib was superior to tofacitinib, peficitinib, and filgotinib. Ritlecitinib was superior to tofacitinib and filgotinib. Brepocitinib was superior to filgotinib (Table 7). Based on SUCRA values, ritlecitinib had the highest value (92.1%), followed by upadacitinib (80.2%), brepocitinib (79.9%), ivarmacitinib (44.8%), tofacitinib (42.2%), peficitinib (36.0%), filgotinib (24.2%), and placebo (0.6%) (Figure 3e; Table 3).

**TABLE 7:** League table for endoscopic improvement.

OR 95%CI							
Brepocitinib	​	​	​	​	​	​	​
6.87 (1.09, 194.07)*	Filgotinib	​	​	​	​	​	​
4.6 (0.57, 141.68)	0.67 (0.22, 1.78)	Ivarmacitinib	​	​	​	​	​
5.62 (0.74, 167.93)	0.82 (0.31, 1.98)	1.23 (0.34, 4.54)	Peficitinib	​	​	​	​
12.41 (2.08, 346.9)*	1.81 (1.26, 2.66)*	2.72 (1.11, 7.77)*	2.22 (1, 5.47)	Placebo	​	​	​
0.78 (0.47, 1.29)	0.11 (0, 0.71)*	0.17 (0.01, 1.37)	0.14 (0, 1.04)	0.06 (0, 0.37)*	Ritlecitinib	​	​
5.04 (0.82, 143.64)	0.74 (0.44, 1.24)	1.11 (0.42, 3.32)	0.9 (0.37, 2.37)	0.41 (0.28, 0.58)*	6.5 (1.05, 183.08)*	Tofacitinib	​
1.47 (0.23, 41.29)	0.21 (0.12, 0.38)*	0.32 (0.12, 1)	0.26 (0.11, 0.71)*	0.12 (0.08, 0.18)*	1.89 (0.3, 52.61)	0.29 (0.16, 0.51)*	Upadacitinib

*Means P < 0.05.

#### Safety

##### AEs

Fifteen articles reported AEs, as illustrated in Supplementary Figure S6a. Closed loops in the network originated solely from within-study comparisons, rendering inconsistency testing unnecessary. The forest plots demonstrated no statistical differences in the incidence of AEs between interventions and placebo (Supplementary Figure S6b). Additionally, the league table indicated a higher risk of AEs for upadacitinib compared to filgotinib (Table 8). Based on SUCRA values, ritlecitinib had the highest value (81.5%), followed by filgotinib (76.3%), tofacitinib (66.7%), brepocitinib (65.0%), placebo (54.7%), upadacitinib (30.9%), ivarmacitinib (26.8%), and peficitinib (18.9%) (Figure 3f; Table 3).

**TABLE 8:** League table for adverse events.

OR 95%CI								
Brepocitinib	​	​	​	​	​	​	​	​
0.99 (0.4, 2.44)	Filgotinib	​	​	​	​	​	​	​
0.6 (0.19, 1.84)	0.61 (0.28, 1.3)	Ivarmacitinib	​	​	​	​	​	​
0.54 (0.17, 1.62)	0.54 (0.25, 1.12)	0.89 (0.32, 2.46)	Peficitinib	​	​	​	​	​
0.85 (0.35, 2.01)	0.85 (0.66, 1.1)	1.4 (0.69, 2.96)	1.57 (0.79, 3.23)	Placebo	​	​	​	​
0.59 (0.17, 2.11)	0.6 (0.23, 1.57)	0.99 (0.3, 3.25)	1.11 (0.35, 3.58)	0.7 (0.27, 1.78)	Prednisolone	​	​	​
1.2 (0.76, 1.91)	1.21 (0.49, 3)	2.01 (0.65, 6.28)	2.24 (0.74, 6.89)	1.42 (0.6, 3.41)	2.02 (0.57, 7.29)	Ritlecitinib	​	​
0.91 (0.37, 2.23)	0.92 (0.66, 1.29)	1.52 (0.72, 3.3)	1.7 (0.83, 3.61)	1.08 (0.87, 1.35)	1.54 (0.62, 3.85)	0.76 (0.31, 1.85)	Tofacitinib	​
0.7 (0.29, 1.7)	0.71 (0.52, 0.97)*	1.17 (0.56, 2.51)	1.31 (0.64, 2.76)	0.83 (0.69, 1)	1.18 (0.46, 3.07)	0.58 (0.24, 1.41)	0.77 (0.58, 1.03)	Upadacitinib

*Means P < 0.05.

##### SAEs

Fifteen articles reported SAEs, as illustrated in Supplementary Figure S7a. As closed loops stemmed solely from within-study comparisons, no inconsistency testing was conducted. Both forest plots and league tables indicated no statistical differences in the incidence of SAEs between interventions or between interventions and placebo (Supplementary Figure S7b; Table 9). Based on SUCRA values, tofacitinib had the highest value (72.3%), followed by peficitinib (60.0%), upadacitinib (59.0%), placebo (49.1%), filgotinib (44.9%), brepocitinib (40.1%), ritlecitinib (35.5%), and ivarmacitinib (35.3%) (Figure 3g; Table 3).

**TABLE 9:** League table for serious adverse events.

OR 95%CI								
Brepocitinib	​	​	​	​	​	​	​	​
1.39 (0.17, 38.73)	Filgotinib	​	​	​	​	​	​	​
0.86 (0.02, 40.18)	0.61 (0.02, 5.46)	Ivarmacitinib	​	​	​	​	​	​
1.89 (0.11, 67.62)	1.34 (0.16, 7.16)	2.2 (0.12, 86.41)	Peficitinib	​	​	​	​	​
1.51 (0.2, 40.18)	1.08 (0.64, 1.94)	1.76 (0.22, 50.43)	0.81 (0.17, 6.35)	Placebo	​	​	​	​
1.74 (0.03, 202.39)	1.14 (0.03, 44.02)	2.01 (0.03, 236.67)	0.89 (0.02, 52.48)	1.04 (0.03, 38.75)	Prednisolone	​	​	​
0.89 (0.28, 2.8)	0.64 (0.02, 5.04)	1.03 (0.02, 49.14)	0.47 (0.01, 8.18)	0.59 (0.02, 4.26)	0.51 (0, 35.2)	Ritlecitinib	​	​
2.02 (0.25, 54.54)	1.45 (0.7, 3.06)	2.34 (0.27, 68.84)	1.08 (0.21, 8.87)	1.33 (0.81, 2.13)	1.27 (0.04, 46.17)	2.25 (0.29, 60.31)	Tofacitinib	​
1.67 (0.21, 45.73)	1.2 (0.63, 2.37)	1.94 (0.23, 56.35)	0.9 (0.18, 7.21)	1.1 (0.76, 1.58)	1.05 (0.03, 40.36)	1.87 (0.25, 50.23)	0.83 (0.46, 1.53)	Upadacitinib

*Means p < 0.05.

##### AEs leading to treatment discontinuation

Fourteen articles reported AEs leading to treatment discontinuation, as illustrated in Supplementary Figure S8a. Closed loops in the network originated solely from within-study comparisons, rendering inconsistency testing unnecessary. The forest plots demonstrated that compared to placebo, upadacitinib [OR = 0.592, 95% CI (0.399, 0.885)] was associated with a significantly reduced likelihood of AEs leading to treatment discontinuation. All other interventions showed no statistical differences compared to placebo (Supplementary Figure S8b). Additionally, the league table revealed no statistical differences in pairwise comparisons between various JAK inhibitors (Table 10). Based on SUCRA values, upadacitinib had the highest value (81.3%), followed by tofacitinib (62.8%), filgotinib (60.3%), ivarmacitinib (56.2%), brepocitinib (46.8%), placebo (40%), peficitinib (37.1%), and ritlecitinib (15.4%) (Figure 3h; Table 3).

**TABLE 10:** League table for adverse events leading to treatment discontinuation.

OR 95%CI							
Brepocitinib	​	​	​	​	​	​	​
1.54 (0.18, 49.43)	Filgotinib	​	​	​	​	​	​
1.58 (0.03, 104.48)	0.99 (0.03, 13.9)	Ivarmacitinib	​	​	​	​	​
0.97 (0.07, 35.07)	0.63 (0.12, 2.37)	0.63 (0.03, 23.51)	Peficitinib	​	​	​	​
1.21 (0.15, 37.9)	0.79 (0.48, 1.35)	0.79 (0.06, 24.6)	1.25 (0.37, 6.01)	Placebo	​	​	​
0.44 (0.13, 1.29)	0.29 (0.01, 2.08)	0.28 (0, 14.17)	0.45 (0.01, 5.45)	0.37 (0.01, 2.43)	Ritlecitinib	​	​
1.59 (0.19, 50.13)	1.03 (0.5, 2.15)	1.04 (0.08, 33.36)	1.65 (0.43, 8.49)	1.31 (0.77, 2.17)	3.59 (0.5, 106.14)	Tofacitinib	​
2.05 (0.25, 65.34)	1.34 (0.7, 2.58)	1.34 (0.1, 42.23)	2.12 (0.59, 10.68)	1.69 (1.13, 2.51)*	4.63 (0.66, 138.27)	1.29 (0.68, 2.5)	Upadacitinib

*Means p < 0.05.

##### Infections

Fourteen articles reported infections, as illustrated in Supplementary Figure S9a. Closed loops in the network originated solely from within-study comparisons, rendering inconsistency testing unnecessary. The forest plots demonstrated that compared to placebo, brepocitinib [OR = 6.75, 95% CI (1.12, 180)] and upadacitinib [OR = 1.72, 95% CI (1.18, 2.58)] were associated with a significantly increased risk of infections. The differences between all other interventions and placebo were not statistically significant (Supplementary Figure S9b). Additionally, the league table suggested that brepocitinib was associated with a higher risk of infections compared to ivarmacitinib and ritlecitinib (Table 11). Based on SUCRA values, ivarmacitinib had the highest value (80.3%), followed by placebo (68.4%), peficitinib (65.5%), filgotinib (52.8%), tofacitinib (44.9%), ritlecitinib (26.7%), upadacitinib (26.6%), brepocitinib (3.5%) (Figure 3i; Table 3).

**TABLE 11:** League table for infections.

OR 95%CI								
Brepocitinib	​	​	​	​	​	​	​	​
5.77 (0.92, 156.16)	Filgotinib	​	​	​	​	​	​	​
12 (1.16, 387.76)*	2.04 (0.47, 7.67)	Ivarmacitinib	​	​	​	​	​	​
7.48 (0.89, 220.9)	1.28 (0.42, 3.47)	0.63 (0.12, 3.49)	Peficitinib	​	​	​	​	​
6.75 (1.12, 179.79)*	1.17 (0.84, 1.65)	0.58 (0.16, 2.42)	0.91 (0.36, 2.66)	Placebo	​	​	​	​
19.45 (0.85, 1848.62)	2.93 (0.28, 88.5)	1.5 (0.1, 55.22)	2.34 (0.19, 77.31)	2.48 (0.25, 73.69)	Prednisolone	​	​	​
2.17 (1.08, 4.58)*	0.38 (0.01, 2.49)	0.18 (0.01, 1.93)	0.29 (0.01, 2.54)	0.32 (0.01, 2.05)	0.11 (0, 2.64)	Ritlecitinib	​	​
5.25 (0.84, 141.87)	0.91 (0.57, 1.46)	0.45 (0.12, 1.95)	0.71 (0.26, 2.17)	0.78 (0.55, 1.08)	0.31 (0.01, 3.02)	2.41 (0.36, 65.65)	Tofacitinib	​
3.92 (0.61, 106.59)	0.68 (0.4, 1.13)	0.33 (0.09, 1.48)	0.53 (0.19, 1.65)	0.58 (0.39, 0.85)*	0.23 (0.01, 2.42)	1.8 (0.27, 49.98)	0.75 (0.45, 1.24)	Upadacitinib

*Means p < 0.05.

### Subgroup analysis

Six studies involving three JAK inhibitors were included in the CD subgroup. For clinical remission, the league table indicated that filgotinib was superior to tofacitinib [OR = 2.69, 95% CI (1.1, 6.91)], while tofacitinib [OR = 0.49, 95% CI (0.29, 0.82)] was inferior to upadacitinib (Supplementary Table S1). SUCRA values ranked filgotinib (90.5%) highest, followed by upadacitinib (75.5%), tofacitinib (24.7%), and placebo (9.2%) (Supplementary Figure S10; Table 12). Regarding clinical response, tofacitinib [OR = 0.4, 95% CI (0.18, 0.9)] performed worse than upadacitinib (Supplementary Table S2). SUCRA values ranked upadacitinib (86.9%) highest, followed by filgotinib (76.6%), placebo (19%), and tofacitinib (17.4%) (Supplementary Figure S11; Table 12). No statistically significant differences were observed among interventions for endoscopic remission (Supplementary Table S3). Based on SUCRA values, upadacitinib (95%) ranked highest, followed by filgotinib (51.3%) and placebo (3.6%) (Supplementary Figure S12; Table 12). For endoscopic response, filgotinib [OR = 0.28, 95% CI (0.1, 0.88)] was inferior to upadacitinib (Supplementary Table S4). SUCRA values ranked upadacitinib (99.2%) highest, followed by filgotinib (48.5%) and placebo (2.3%) (Supplementary Figure S13; Table 12). No significant differences were detected for AEs (Supplementary Table S5). Tofacitinib (84.5%) exhibited the lowest risk based on SUCRA, followed by placebo (50.6%), filgotinib (50.1%), and upadacitinib (14.8%) (Supplementary Figure S14; Table 12). For SAEs, no significant differences were observed (Supplementary Table S6). Placebo (75.4%) demonstrated the lowest risk per SUCRA, followed by upadacitinib (58.2%), filgotinib (42.9%), and tofacitinib (23.5%) (Supplementary Figure S15; Table 12). No significant differences emerged for AEs leading to treatment discontinuation (Supplementary Table S7). SUCRA values indicated the lowest risk for tofacitinib (63.4%), followed by filgotinib (61.9%), upadacitinib (40.7%), and placebo (34%) (Supplementary Figure S16; Table 12). For infections, tofacitinib [OR = 0.22, 95% CI (0.06, 0.76)] posed a lower risk than upadacitinib (Supplementary Table S8). SUCRA values ranked tofacitinib (63.4%) lowest in risk, followed by filgotinib (61.9%), upadacitinib (40.7%), and placebo (34%) (Supplementary Figure S17; Table 12).

**TABLE 12:** NMA subgroup analysis: SUCRA ranking for Crohn’s disease.

Treatment	Clinical remission (%)	Clinical response (%)	Endoscopic remission (%)	Endoscopic response (%)	Adverse events (%)	Serious adverse events (%)	Adverse events leading to treatment discontinuation (%)	Infections (%)
Filgotinib	90.5	76.6	51.3	48.5	50.1	42.9	61.9	49.1
Upadacitinib	75.5	86.9	95	99.2	14.8	58.2	40.7	3
Tofacitinib	24.7	17.4	-	-	84.5	23.5	63.4	93.1
Placebo	9.2	19	3.6	2.3	50.6	75.4	34	54.8

Nine studies evaluating seven JAK inhibitors were included in the UC subgroup. For clinical remission, filgotinib [OR = 0.19, 95% CI (0.09, 0.38)] and tofacitinib [OR = 0.3, 95% CI (0.14, 0.6)] were inferior to upadacitinib (Supplementary Table S9). SUCRA values ranked ritlecitinib (80.7%) highest, followed by upadacitinib (80.1%), ivarmacitinib (69.4%), brepocitinib (63.3%), peficitinib (45.4%), tofacitinib (39.7%), filgotinib (20.1%), and placebo (0.5%) (Supplementary Figure S18; Supplementary Table S10). Regarding clinical response, filgotinib [OR = 0.32, 95% CI (0.21, 0.48)], ivarmacitinib [OR = 0.29, 95% CI (0.13, 0.7)], peficitinib [OR = 0.24, 95% CI (0.11, 0.51)], and tofacitinib [OR = 0.36, 95% CI (0.24, 0.54)] were all inferior to upadacitinib (Supplementary Table S11). SUCRA values ranked upadacitinib (95.9%) highest, followed by ritlecitinib (78.7%), prednisolone (64.9%), brepocitinib (54.3%), tofacitinib (50.7%), filgotinib (41.0%), ivarmacitinib (37.9%), peficitinib (25.8%), and placebo (0.7%) (Supplementary Figure S19; Supplementary Table S10). No significant differences were observed for endoscopic remission (Supplementary Table S12). SUCRA values ranked upadacitinib (94.3%) highest, followed by filgotinib (52%) and placebo (3.7%) (Supplementary Figure S20; Supplementary Table S10). For endoscopic improvement, brepocitinib [OR = 6.86, 95% CI (1.14, 165.5)] outperformed filgotinib, while filgotinib [OR = 0.22, 95% CI (0.12, 0.38)], peficitinib [OR = 0.26, 95% CI (0.11, 0.71)], and tofacitinib [OR = 0.29, 95% CI (0.17, 0.52)] were inferior to upadacitinib. Filgotinib [OR = 0.11, 95% CI (0, 0.68)] was also inferior to ritlecitinib, and ritlecitinib [OR = 6.43, 95% CI (1.09, 153.67)] outperformed tofacitinib (Supplementary Table S13). SUCRA values ranked ritlecitinib (92.3%) highest, followed by brepocitinib (80.2%), upadacitinib (80.1%), ivarmacitinib (44.7%), tofacitinib (42.2%), peficitinib (35.7%), filgotinib (24.2%), and placebo (0.6%) (Supplementary Figure S21; Supplementary Table S10). No significant differences were detected for AEs (Supplementary Table S14). SUCRA values indicated the lowest risk for ritlecitinib (82.1%), followed by filgotinib (78.5%), brepocitinib (65.7%), tofacitinib (58.9%), placebo (57%), upadacitinib (34.1%), ivarmacitinib (28%), prednisolone (25.7%), and peficitinib (19.9%) (Supplementary Figure S22; Supplementary Table S10). For SAEs, no significant differences emerged (Supplementary Table S15). SUCRA values ranked upadacitinib (76%) lowest in risk, followed by tofacitinib (74.6%), prednisolone (56.6%), peficitinib (54%), filgotinib (41.8%), placebo (41.8%), ritlecitinib (38%), brepocitinib (35.8%), and ivarmacitinib (31.5%) (Supplementary Figure S23; Supplementary Table S10). Regarding AEs leading to treatment discontinuation, filgotinib [OR = 3.37, 95% CI (1.33, 9.05)], peficitinib [OR = 5.28, 95% CI (1.27, 29.21)], ritlecitinib [OR = 11.12, 95% CI (1.49, 359.64)], and tofacitinib [OR = 3.25, 95% CI (1.29, 8.66)] all exhibited higher risks than upadacitinib (Supplementary Table S16). SUCRA values indicated the lowest risk for upadacitinib (95.7%), followed by tofacitinib (58.1%), filgotinib (56%), ivarmacitinib (53.1%), brepocitinib (44.9%), placebo (41.5%), peficitinib (35.6%), and ritlecitinib (15.1%) (Supplementary Figure S24; Supplementary Table S10). For infections, brepocitinib posed a higher risk than ivarmacitinib [OR = 11.94, 95% CI (1.17, 346.8)] and ritlecitinib [OR = 2.18, 95% CI (1.07, 4.61)] (Supplementary Table S17). SUCRA values ranked ivarmacitinib (80.5%) lowest in risk, followed by prednisolone (79.3%), placebo (68.5%), peficitinib (65.8%), filgotinib (50.8%), upadacitinib (38.2%), tofacitinib (37.1%), ritlecitinib (26.5%), and brepocitinib (3.3%) (Supplementary Figure S25; Supplementary Table S10).

### Evaluation of publication bias

The publication bias was assessed via funnel plots for the following items: endoscopic remission, endoscopic response, endoscopic improvement, clinical remission, clinical response, AEs, SAEs, AEs leading to treatment discontinuation, and infections. The results indicated a high probability of publication bias (Supplementary Figures S26–S34).

## Discussion

This study used an innovative NMA methodology to evaluate the relative efficacy and safety of various JAK inhibitors for IBD treatment by incorporating five efficacy outcomes (clinical remission, clinical response, endoscopic remission, endoscopic response, and endoscopic improvement) and four safety outcomes (AEs, SAEs, AEs leading to treatment discontinuation, and infections). This study is the first comprehensive assessment of its kind, providing valuable evidence for clinical decision-making. However, NMAs of malignancy, dyslipidemia, and thrombosis could not be conducted due to limited event data.

According to the results of this study, all of the inhibitors demonstrated superior efficacy compared to placebo. The safety profiles of the remaining inhibitors did not differ significantly from placebo, except for upadacitinib and brepocitinib. Ritlecitinib exhibited the most prominent efficacy in achieving clinical remission, clinical response, and endoscopic improvement. It is a novel dual-target inhibitor that selectively inhibits the JAK3 and TEC kinase families. Studies of its mechanism have shown that JAK3 plays a crucial role in lymphocyte development, proliferation, and functional regulation (Salas et al., 2020). The TEC kinase family regulates multiple immune response signaling pathways and inhibits the cytotoxic functions of CD8^+^ T cells and natural killer cells. Previous studies have confirmed the important role of the JAK3/TEC kinase family in the pathogenesis of IBD (Guan et al., 2021; Lechner et al., 2021; Xu et al., 2019; Hassan-Zahraee et al., 2024). However, ritlecitinib’s potent efficacy may come at the cost of lower safety. This is because ritlecitinib may lead to immunosuppression while exerting its anti-inflammatory effects, increasing the risk of infection and malignancy. Post-marketing surveillance data from the FDA indicate that ritlecitinib requires a black box warning to highlight the potential risks of serious infections (including bacterial, fungal, viral, and opportunistic infections), sudden cardiac death, malignancy (such as lymphoma and lung cancer), and thromboembolic events (Roskoski, 2024). Our analysis yielded consistent results: despite having the lowest overall risk of AEs, ritlecitinib had the highest risk of AEs leading to treatment discontinuation and ranked second in terms of both SAEs and infection risk. These results may be related to the short follow-up period of this study and clinicians’ high sensitivity to the FDA black box warning, which leads to treatment termination upon any adverse reaction. Currently, ritlecitinib is still in clinical development and has not been approved for treating CD or UC. Future, larger-scale clinical trials with longer follow-up periods are needed to more comprehensively assess its risk-benefit ratio. Upadacitinib exhibited the most prominent efficacy in endoscopic remission and endoscopic response, ranking second only to ritlecitinib in clinical response and demonstrating favorable performance in clinical remission. Furthermore, direct comparisons of upadacitinib with filgotinib, another JAK1 inhibitor, revealed that upadacitinib consistently demonstrated superior efficacy across all indicators in league tables of the five efficacy outcome indicators. As a highly selective, new-generation JAK1 inhibitor, upadacitinib exhibits 60-fold selectivity for JAK1 over JAK2 and 100-fold selectivity for JAK1 over JAK3 (Parmentier et al., 2018). This high selectivity enables the precise inhibition of pro-inflammatory signaling pathways and the effective downregulation of various inflammatory factors, including IL-6, IL-10, IL-11, IL-19, IL-20, IL-22, and IFN-α/β/γ. Consequently, intestinal inflammatory responses are significantly improved (Bonelli et al., 2024; Morris et al., 2018). Aguilar et al. observed areas of mucosal involvement that achieved endoscopic remission following treatment with upadacitinib. They found that upadacitinib can target inflammatory fibroblasts and IFN-γ-expressing cytotoxic T cells in mucosal lesions, thereby alleviating intestinal inflammation (Aguilar et al., 2021). A prospective, real-world study evaluated the efficacy of upadacitinib in patients with refractory UC and CD. As early as Week 2, the clinical remission rates for UC and CD patients were 36% and 56.3%, respectively. By Week 8, the clinical response and remission rates for UC patients were 85.2% and 81.5%, respectively, while the corresponding rates for CD patients were 76.5% and 70.6%, respectively. Fecal calprotectin and C-reactive protein levels normalized in 62% and 64% of patients, respectively (Friedberg et al., 2023). Jean-Frédéric Colombel et al.'s study indicated that upadacitinib rapidly alleviated symptoms such as abdominal pain and diarrhea in CD patients within 1 week (Colombel et al., 2024). Regarding the safety of upadacitinib, the current study found that it had poor SUCRA rankings for AEs and infections but better rankings for SAEs and AEs leading to treatment discontinuation. These results suggest that upadacitinib increases the risk of overall AEs, especially infections, but has a lower risk of SAEs and is well tolerated. Major adverse cardiovascular events (MACEs) and venous thromboembolism (VTE) are considered potential risks associated with JAK inhibitors. However, there is currently insufficient evidence to suggest that upadacitinib increases the risk of MACEs and VTE. This may be due to upadacitinib’s low affinity for JAK2/3 (Evangelatos et al., 2023; Nielsen et al., 2023). A study by Lasa et al. found that upadacitinib posed the highest risk of adverse reactions in patients with moderate to severe UC compared to filgotinib, tofacitinib, ozanimod, ustekinumab, and vedolizumab (Lasa et al., 2022). Another meta-analysis of IBD suggested that upadacitinib was more likely to increase the risk of herpes zoster infection than tofacitinib, filgotinib, and ustekinumab (Din et al., 2023). Clinical study data indicate that upadacitinib is generally well tolerated by patients with rheumatoid arthritis, psoriatic arthritis, and UC. Notably, upadacitinib has not been associated with an increased risk of MACEs, VTE, or malignancies (excluding non-melanoma skin cancer) at any dose in patients with UC (Burmester et al., 2023; Vermeire et al., 2023). Subgroup analyses, which were stratified by disease type, demonstrated a high degree of consistency between subgroups and overall analyses for most outcomes. These findings support the broad efficacy and safety of JAK inhibitors in IBD. Regarding efficacy endpoints—including clinical remission, clinical response, and endoscopic improvement—the overall analysis showed that JAK inhibitors (e.g., ritlecitinib, upadacitinib, and brepocitinib) were significantly more effective than placebo, as indicated by higher SUCRA values. This trend was further corroborated in both the CD and UC subgroups, where these agents also exhibited superior performance. Ritlecitinib notably achieved the highest SUCRA value for endoscopic improvement in the overall analysis (92.1%) and demonstrated comparable excellence in the UC subgroup (92.3%), suggesting a potential specific advantage for endoscopic outcomes in UC. Upadacitinib performed consistently well across multiple endpoints in both the overall and subgroup analyses, supporting its role as a highly effective therapeutic option for IBD. In terms of safety, no significant differences were observed between JAK inhibitors and placebo in the incidence of AEs or SAEs, a finding consistent across subgroups. However, the overall analysis revealed an increased risk of infections with brepocitinib and upadacitinib. This finding was further substantiated in the UC subgroup, where brepocitinib showed a significantly higher infection risk compared to other agents (OR = 11.94). Overall trends were generally consistent, but subgroup analyses revealed heterogeneity in certain outcomes. For instance, filgotinib ranked highest for clinical remission in the CD subgroup (SUCRA: 90.5%), outperforming tofacitinib. However, its overall SUCRA was only 33.9%. This discrepancy may be due to the small sample size in the CD subgroup (only six studies) or to the distinct pathophysiology of CD, which is characterized by transmural inflammation and fibrosis. In this context, filgotinib’s JAK1-selective inhibition may exert more targeted efficacy. The overall analysis, which encompassed a broader population, may have diluted this effect. Additionally, the CD subgroup showed a significantly lower risk of infection with tofacitinib than with upadacitinib (OR = 0.22), a difference that was not observed in the overall analysis. This may reflect the heightened infection susceptibility of CD patients, who often present with penetrating complications and malnutrition. Tofacitinib’s relatively broad JAK inhibition might provide more balanced immunomodulation, an effect potentially masked in the mixed population of the overall analysis.

In summary, we believe that upadacitinib can be considered a first-line treatment for patients with moderate to severe IBD, given its excellent efficacy, reduced risk of SAEs, and favorable tolerability profile. Patients should receive vaccinations, especially the herpes zoster vaccine, before using upadacitinib to reduce the risk of infection. This study is the first to identify the tremendous potential of ritlecitinib in terms of efficacy and clarify the advantages of upadacitinib in terms of the efficacy-safety balance. However, because the study has limitations, the results require careful interpretation. First, this study only collected induction phase data from the included studies. Consequently, the short follow-up period and insufficient sample size hindered the analysis of certain outcome indicators. This led to an overestimation of some drugs’ efficacy and an underestimation of the risk of adverse reactions. Future studies must be conducted with longer follow-up periods to ensure more accurate comparisons between studies. Second, many studies had inconsistent statistical endpoint times and different dose regimens. Additionally, some patients had a history of biological agent treatment. These factors may introduce heterogeneity. Third, these treatments were not evaluated from a cost perspective, which is significant for healthcare providers, payers, and patients when making clinical decisions. Therefore, future studies should prioritize cost-effectiveness assessments. Additional high-quality, head-to-head trials and real-world studies will be required to validate and extend our conclusions.

## Conclusion

This NMA suggests that upadacitinib offers a favorable balance of efficacy and tolerability in the treatment of IBD, positioning it as a preferred JAK inhibitor in the current landscape. Although ritlecitinib demonstrated significant efficacy, its associated risk of SAEs requires careful consideration of the risks and benefits in clinical practice. Further long-term studies are needed to optimize treatment strategies involving JAK inhibitors.

## Data Availability

The original contributions presented in the study are included in the article/Supplementary Material, further inquiries can be directed to the corresponding author.
