# Supplementary material for: Safety and efficacy of different JAK inhibitors in the treatment of inflammatory bowel disease: a network meta-analysis
Source: Front Pharmacol. 2026 Jan 27;16:1699928. doi: 10.3389/fphar.2025.1699928 (PMC12886027; doi:10.3389/fphar.2025.1699928)
Supplement: Supplementary file 1 [file Supplementaryfile1.docx]

**Search strategies**

Pubmed

| No. | Query | Results |
| --- | --- | --- |
| #1 | (Inflammatory Bowel Diseases[MeSH Terms]) OR (((((((((((((((((((((((((Inflammatory Bowel Diseases[Title/Abstract]) OR (Inflammatory Bowel Disease[Title/Abstract])) OR (Bowel Diseases, Inflammatory[Title/Abstract])) OR (Colitis, Ulcerative[Title/Abstract])) OR (Idiopathic Proctocolitis[Title/Abstract])) OR (Ulcerative Colitis[Title/Abstract])) OR (Colitis Gravis[Title/Abstract])) OR (Inflammatory Bowel Disease, Ulcerative Colitis Type[Title/Abstract])) OR (Crohn Disease[Title/Abstract])) OR (Crohn's Enteritis[Title/Abstract])) OR (Regional Enteritis[Title/Abstract])) OR (Crohn's Disease[Title/Abstract])) OR (Crohns Disease[Title/Abstract])) OR (Inflammatory Bowel Disease 1[Title/Abstract])) OR (Enteritis, Granulomatous[Title/Abstract])) OR (Granulomatous Enteritis[Title/Abstract])) OR (Enteritis, Regional[Title/Abstract])) OR (Ileocolitis[Title/Abstract])) OR (Colitis, Granulomatous[Title/Abstract])) OR (Granulomatous Colitis[Title/Abstract])) OR (Ileitis, Terminal[Title/Abstract])) OR (Terminal Ileitis[Title/Abstract])) OR (Ileitis, Regional[Title/Abstract])) OR (Regional Ileitides[Title/Abstract])) OR (Regional Ileitis[Title/Abstract])) | 151097 |
| #2 | (Janus Kinase Inhibitors[MeSH Terms]) OR (((((((((((((((Janus Kinase Inhibitors[Title/Abstract]) OR (Inhibitors, Janus Kinase[Title/Abstract])) OR (Kinase Inhibitors, Janus[Title/Abstract])) OR (JAK Inhibitors[Title/Abstract])) OR (Inhibitors, JAK[Title/Abstract])) OR (Janus Kinase Inhibitor[Title/Abstract])) OR (Inhibitor, Janus Kinase[Title/Abstract])) OR (Kinase Inhibitor, Janus[Title/Abstract])) OR (JAK Inhibitor[Title/Abstract])) OR (Inhibitor, JAK[Title/Abstract])) OR (ruxolitinib[Title/Abstract])) OR (tofacitinib[Title/Abstract])) OR (Baricitinib[Title/Abstract])) OR (ritlecitinib[Title/Abstract])) OR (delgocitinib[Title/Abstract])) | 13262 |
| #3 | (randomized controlled trial[Publication Type]) OR (randomized[Title/Abstract]) OR (placebo[Title/Abstract]) | 1154445 |
| #4 | #1 AND #2 AND #3 | 244 |

Cochrane

| No. | Query | Results |
| --- | --- | --- |
| #1 | Inflammatory Bowel Diseases | 2879 |
| #2 | (Inflammatory Bowel Diseases):ti,ab,kw OR (Bowel Diseases, Inflammatory):ti,ab,kw OR (Colitis, Ulcerative):ti,ab,kw OR (Idiopathic Proctocolitis):ti,ab,kw OR (Ulcerative Colitis):ti,ab,kw | 7764 |
| #3 | (Colitis Gravis):ti,ab,kw OR (Inflammatory Bowel Disease, Ulcerative Colitis Type):ti,ab,kw OR (Crohn Disease):ti,ab,kw OR (Crohn's Enteritis):ti,ab,kw OR (Regional Enteritis):ti,ab,kw | 6055 |
| #4 | (Crohn's Disease):ti,ab,kw OR (Crohns Disease):ti,ab,kw OR (Inflammatory Bowel Disease 1):ti,ab,kw OR (Enteritis, Granulomatous):ti,ab,kw OR (Granulomatous Enteritis):ti,ab,kw | 7889 |
| #5 | (Enteritis, Regional):ti,ab,kw OR (Ileocolitis):ti,ab,kw OR (Colitis, Granulomatous):ti,ab,kw OR (Granulomatous Colitis):ti,ab,kw OR (Ileitis, Terminal):ti,ab,kw | 145 |
| #6 | (Terminal Ileitis):ti,ab,kw OR (Ileitis, Regional):ti,ab,kw OR (Regional Ileitides):ti,ab,kw OR (Regional Ileitis):ti,ab,kw | 33 |
| #7 | #1 OR #2 OR #3 OR #4 OR #5 OR #6 | 13137 |
| #8 | MeSH descriptor: [Janus Kinase Inhibitors] explode all trees | 222 |
| #9 | (Janus Kinase Inhibitors):ti,ab,kw OR (Inhibitors, Janus Kinase):ti,ab,kw OR (Kinase Inhibitors, Janus):ti,ab,kw OR (JAK Inhibitors):ti,ab,kw NOT (Inhibitors, JAK):ti,ab,kw | 676 |
| #10 | (Janus Kinase Inhibitor):ti,ab,kw OR (Inhibitor, Janus Kinase):ti,ab,kw OR (Kinase Inhibitor, Janus):ti,ab,kw OR (JAK Inhibitor):ti,ab,kw OR (Inhibitor, JAK):ti,ab,kw | 2299 |
| #11 | (ruxolitinib):ti,ab,kw OR (tofacitinib):ti,ab,kw OR (Baricitinib):ti,ab,kw OR (ritlecitinib):ti,ab,kw OR (delgocitinib):ti,ab,kw | 2039 |
| #12 | #8 OR #9 OR #10 OR #11 | 3338 |
| #13 | #7 AND #12 | 528 |

Web of science

| No. | Query | Results |
| --- | --- | --- |
| #1 | TS=(Inflammatory Bowel Diseases) OR TS=(Inflammatory Bowel Disease) OR TS=(Bowel Diseases, Inflammatory) OR TS=(Colitis, Ulcerative) OR TS=(Idiopathic Proctocolitis) OR TS=(Ulcerative Colitis) OR TS=(Colitis Gravis) OR TS=(Inflammatory Bowel Disease, Ulcerative Colitis Type) OR TS=(Crohn Disease) OR TS=(Crohn's Enteritis) OR TS=(Regional Enteritis) OR TS=(Crohn's Disease) OR TS=(Crohns Disease) OR TS=(Inflammatory Bowel Disease 1) OR TS=(Enteritis, Granulomatous) OR TS=(Granulomatous Enteritis) OR TS=(Enteritis, Regional) OR TS=(Ileocolitis) OR TS=(Colitis, Granulomatous) OR TS=(Granulomatous Colitis) OR TS=(Ileitis, Terminal) OR TS=(Terminal Ileitis) OR TS=(Ileitis, Regional) OR TS=(Regional Ileitides) OR TS=(Regional Ileitis) and Preprint Citation Index (Troubleshooting - Database) | 129899 |
| #2 | TS=(random) OR TS=(placebo) OR TS=(double-blind) OR TS=(randomized controlled trial) OR TS=(randomized) and Preprint Citation Index (Troubleshooting - Database) | 1461775 |
|  | S=(Janus Kinase Inhibitors) OR TS=(Inhibitors, Janus Kinase) OR TS=(Kinase Inhibitors, Janus) OR TS=(JAK Inhibitors) OR TS=(Inhibitors, JAK) OR TS=(Janus Kinase Inhibitor) OR TS=(Inhibitor, Janus Kinase) OR TS=(Kinase Inhibitor, Janus) OR TS=(JAK Inhibitor) OR TS=(Inhibitor, JAK) OR TS=(ruxolitinib) OR TS=(tofacitinib) OR TS=(Baricitinib) OR TS=(ritlecitinib) OR TS=(delgocitinib) and Preprint Citation Index (Troubleshooting - Database) | 21555 |
| #3 | #3 AND #2 AND #1 and Preprint Citation Index (Troubleshooting - Database) | 592 |

Embase

| No. | Query | Results |
| --- | --- | --- |
| #49 | #27 AND #44 AND #48 | 750 |
| #48 | #45 OR #46 OR #47 | 892906 |
| #47 | 'double-blind':ab,ti | 230456 |
| #46 | 'placebo':ab,ti | 376357 |
| #45 | 'random':ab,ti | 456270 |
| #44 | #28 OR #29 OR #30 OR #31 OR #32 OR #33 OR #34 OR #35 OR #36 OR #37 OR #38 OR #39 OR #40 OR #41 OR #42 OR #43 | 36589 |
| #43 | 'delgocitinib':ab,ti | 82 |
| #42 | 'ritlecitinib':ab,ti | 99 |
| #41 | 'baricitinib':ab,ti | 21 |
| #40 | 'tofacitinib':ab,ti | 0 |
| #39 | 'ruxolitinib':ab,ti | 6316 |
| #38 | 'inhibitor, jak':ab,ti | 48 |
| #37 | 'jak inhibitor':ab,ti | 3650 |
| #36 | 'kinase inhibitor, janus':ab,ti | 0 |
| #35 | 'inhibitor, janus kinase':ab,ti | 1 |
| #34 | 'janus kinase inhibitor':ab,ti | 1755 |
| #33 | 'inhibitors, jak':ab,ti | 105 |
| #32 | 'jak inhibitors':ab,ti | 3962 |
| #31 | 'kinase inhibitors, janus':ab,ti | 2 |
| #30 | 'inhibitors, janus kinase':ab,ti | 27 |
| #29 | 'janus kinase inhibitors':ab,ti | 1490 |
| #28 | 'janus kinase inhibitor'/exp | 35510 |
| #27 | #1 OR #2 OR #3 OR #4 OR #5 OR #6 OR #7 OR #8 OR #9 OR #10 OR #11 OR #12 OR #13 OR #14 OR #15 OR #16 OR #17 OR #18 OR #19 OR #20 OR #21 OR #22 OR #23 OR #24 OR #25 OR #26 | 245198 |
| #26 | 'regional ileitis':ab,ti | 290 |
| #25 | 'regional ileitides':ab,ti | 0 |
| #24 | 'ileitis, regional':ab,ti | 6 |
| #23 | 'terminal ileitis':ab,ti | 754 |
| #22 | 'ileitis, terminal':ab,ti | 2 |
| #21 | 'granulomatous colitis':ab,ti | 548 |
| #20 | 'colitis, granulomatous':ab,ti | 12 |
| #19 | 'ileocolitis':ab,ti | 778 |
| #18 | 'enteritis, regional':ab,ti | 5 |
| #17 | 'granulomatous enteritis':ab,ti | 231 |
| #16 | 'enteritis, granulomatous':ab,ti | 3 |
| #15 | 'crohns disease':ab,ti | 1115 |
| #14 | 'inflammatory bowel disease 1':ab,ti | 168 |
| #13 | 'crohns disease':ab,ti | 1115 |
| #12 | 'regional enteritis':ab,ti | 969 |
| #11 | 'crohns enteritis':ab,ti | 0 |
| #10 | 'crohn disease':ab,ti | 6124 |
| #9 | 'inflammatory bowel disease, ulcerative colitis type':ab,ti | 2 |
| #8 | 'colitis gravis':ab,ti | 6 |
| #7 | 'ulcerative colitis':ab,ti | 83678 |
| #6 | 'idiopathic proctocolitis':ab,ti | 44 |
| #5 | 'colitis, ulcerative':ab,ti | 140 |
| #4 | 'bowel diseases, inflammatory':ab,ti | 15 |
| #3 | 'inflammatory bowel disease':ab,ti | 96498 |
| #2 | 'inflammatory bowel diseases':ab,ti | 18409 |
| #1 | 'inflammatory bowel disease'/exp | 214387 |
