# Supplementary material for: Safety and efficacy of different JAK inhibitors in the treatment of inflammatory bowel disease: a network meta-analysis
Source: Front Pharmacol. 2026 Jan 27;16:1699928. doi: 10.3389/fphar.2025.1699928 (PMC12886027; doi:10.3389/fphar.2025.1699928)
Supplement: Supplementary file 2 [file Supplementaryfile2.docx]

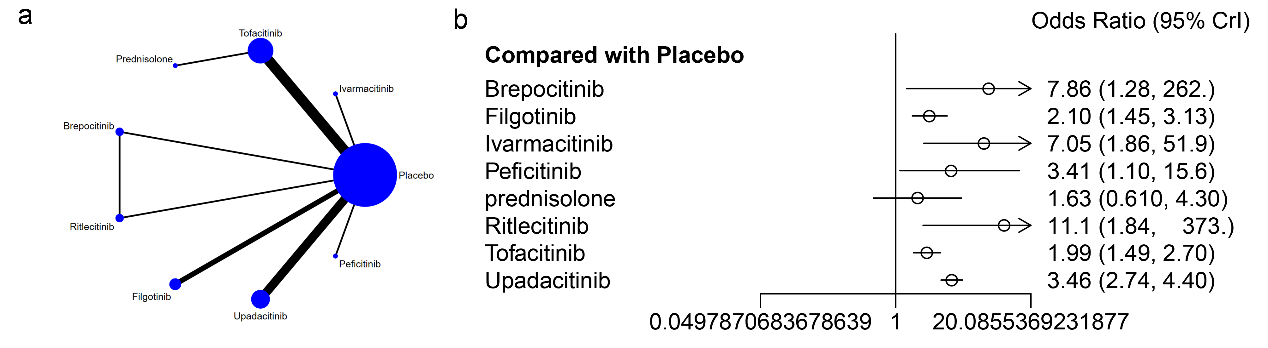


Figure S1 a: Network plot of clinical remission, b: Forest plot of clinical response


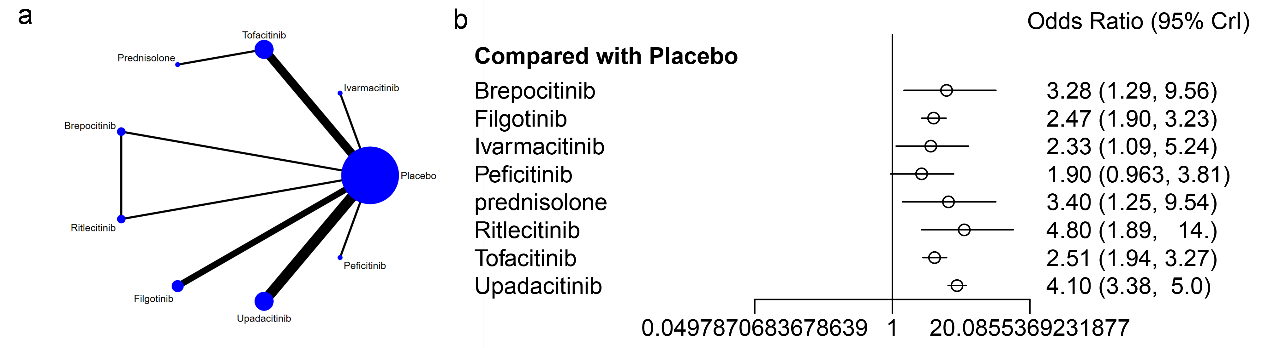


Figure S2 a: Network plot of clinical response, b: Forest plot of clinical response


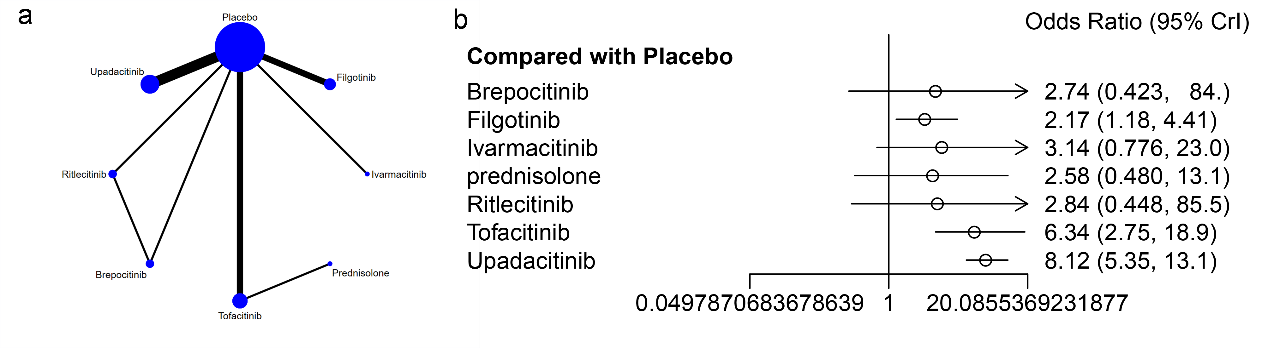


Figure S3 a: Network plot of endoscopic remission, b: Forest plot of endoscopic remission


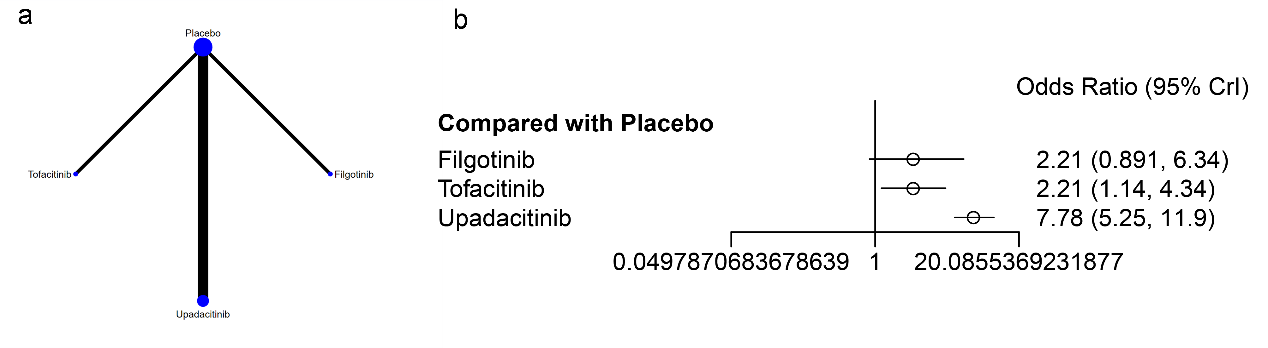


Figure S4 a: Network plot of endoscopic response, b: Forest plot of endoscopic response


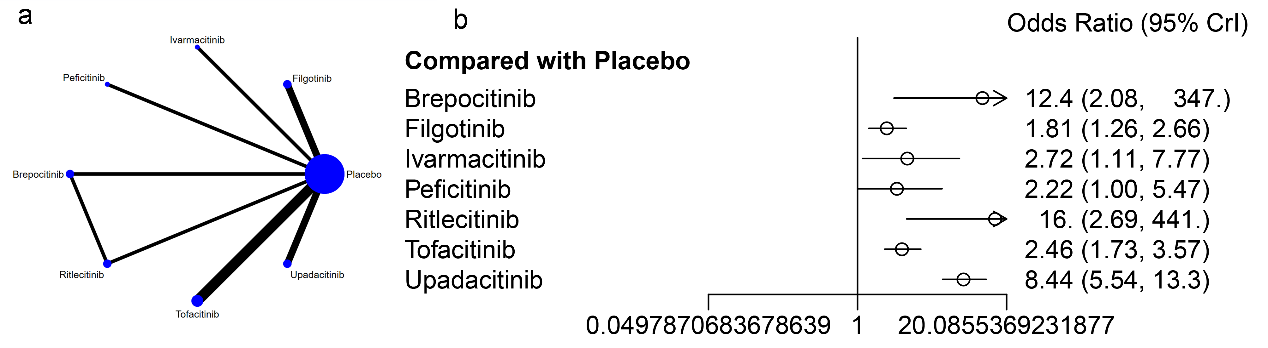


Figure S5 a: Network plot of endoscopic improvement, b: Forest plot of endoscopic improvement


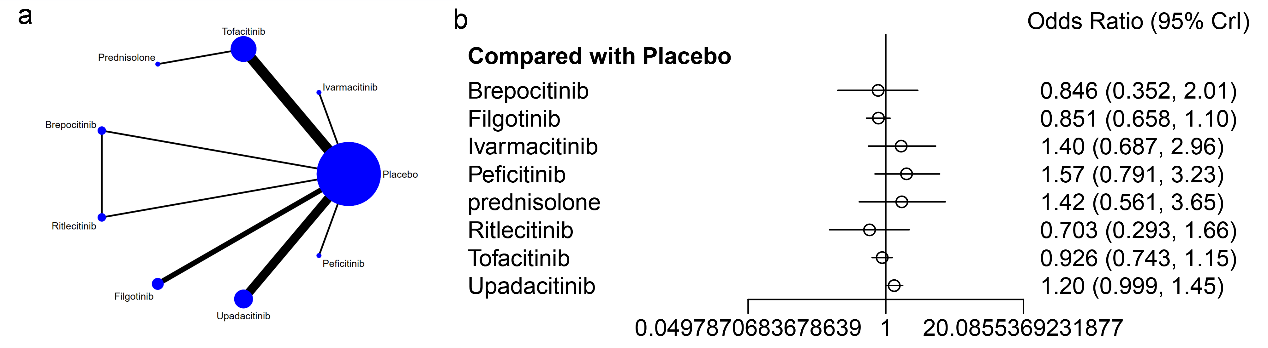


Figure S6 a: Network plot of adverse events, b: Forest plot of adverse events


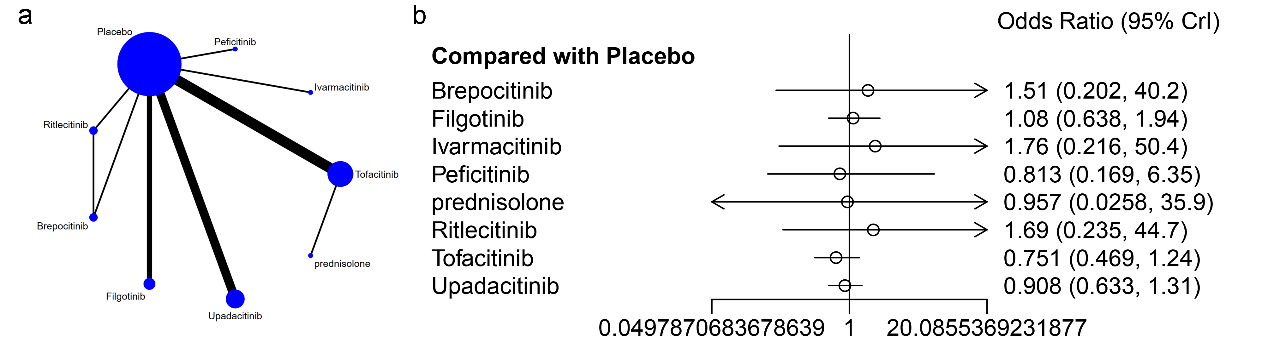


Figure S7 a: Network plot of serious adverse events, b: Forest plot of serious adverse events


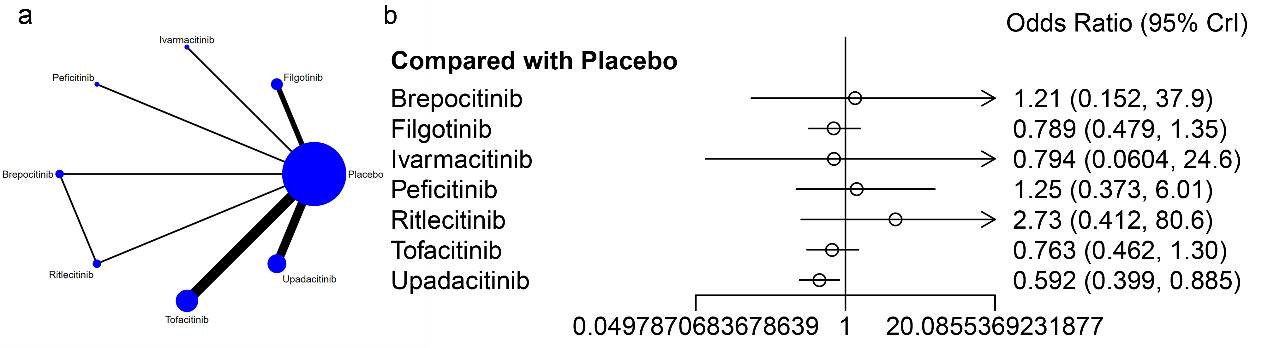


Figure S8 a: Network plot of adverse events leading to treatment discontinuation, b: Forest plot of adverse events leading to treatment discontinuation


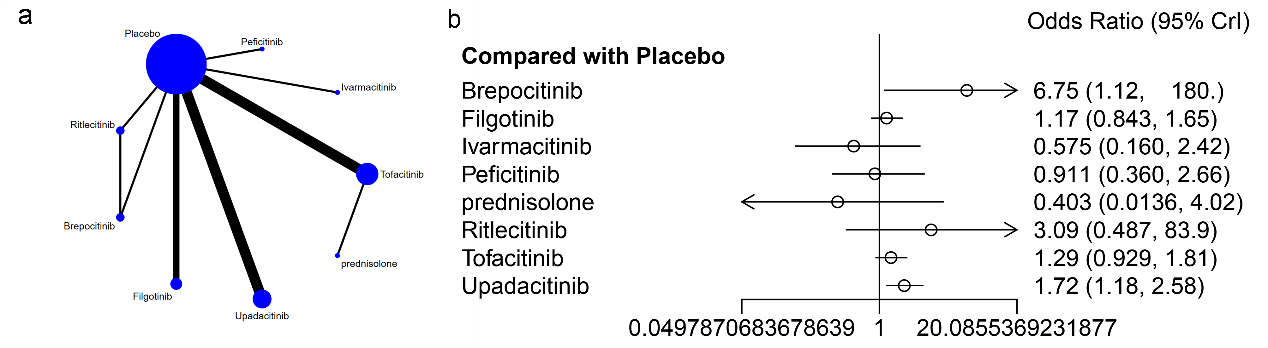


Figure S9 a: Network plot of infections, b: Forest plot of infections


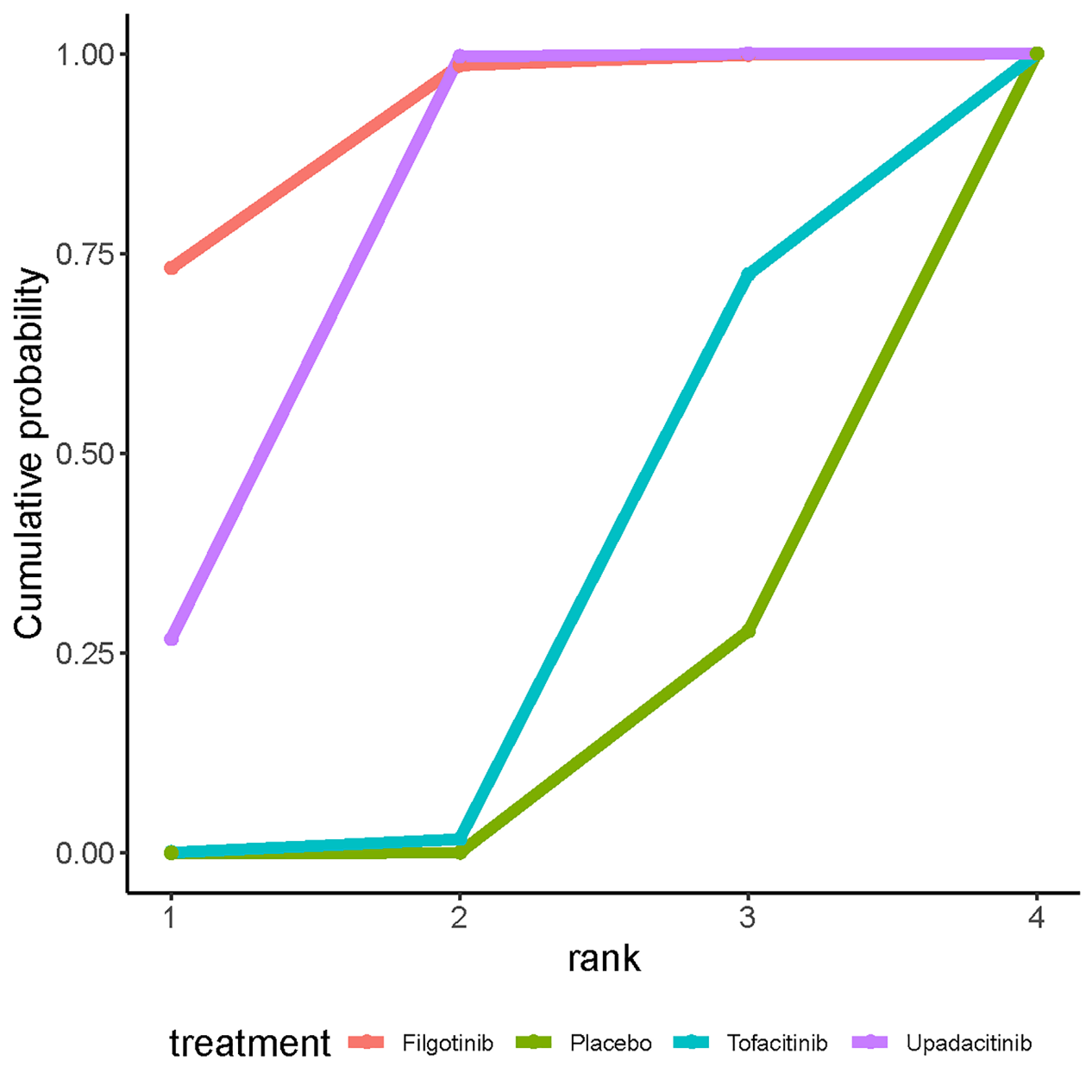


Figure S10 Line chart of clinical remission in the Crohn's disease subgroup


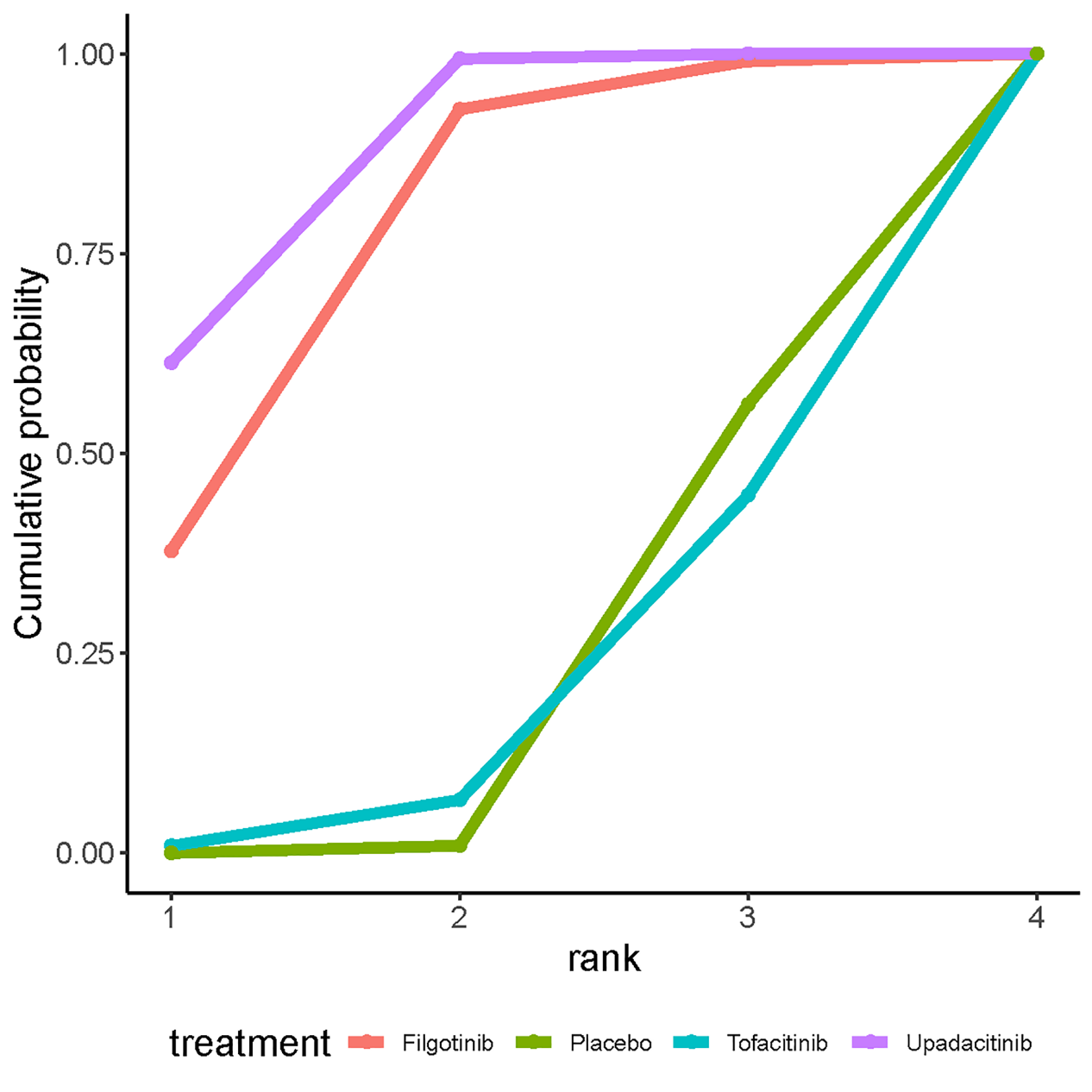


Figure S11 Line chart of clinical response in the Crohn's disease subgroup


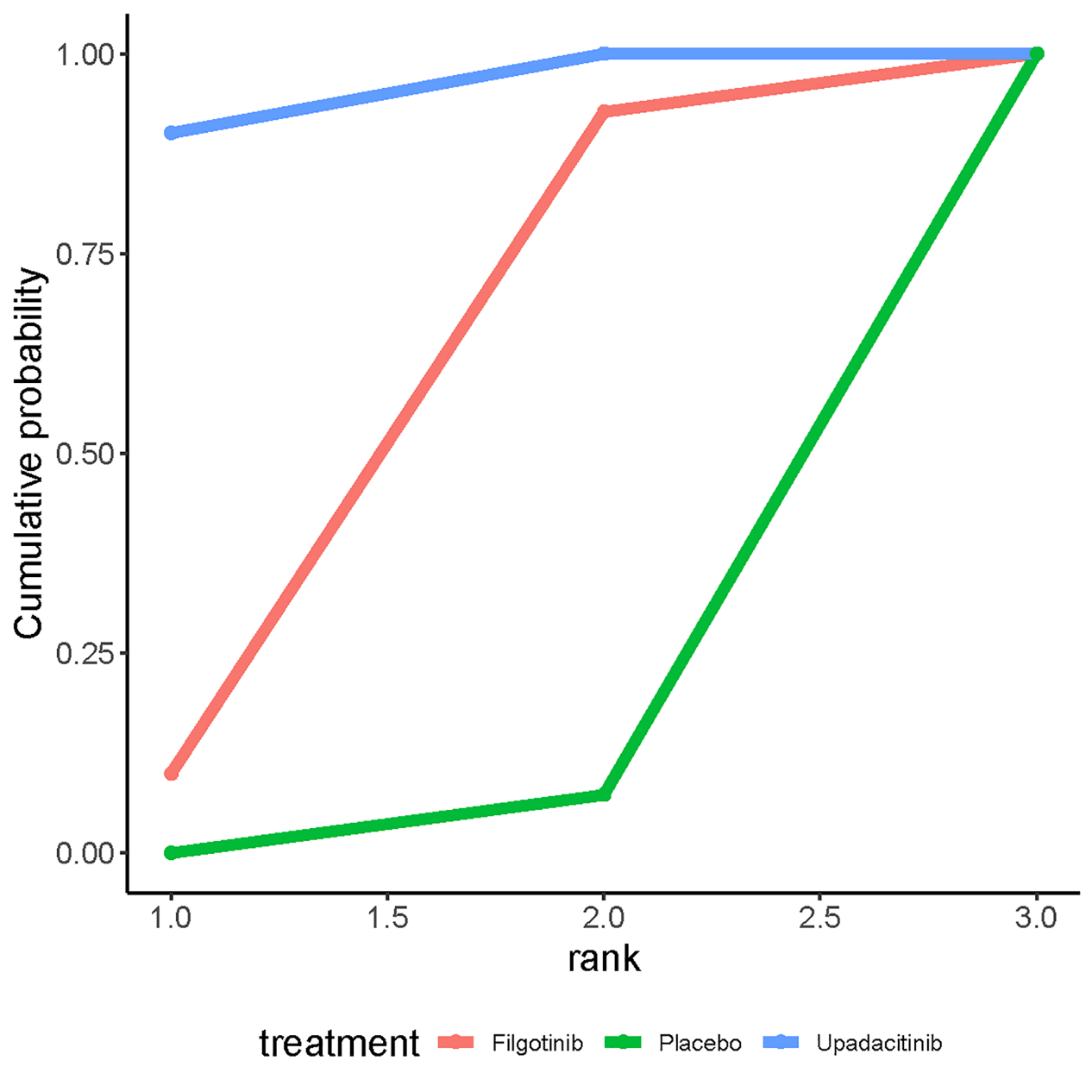


Figure S12 Line chart of endoscopic remission in the Crohn's disease subgroup


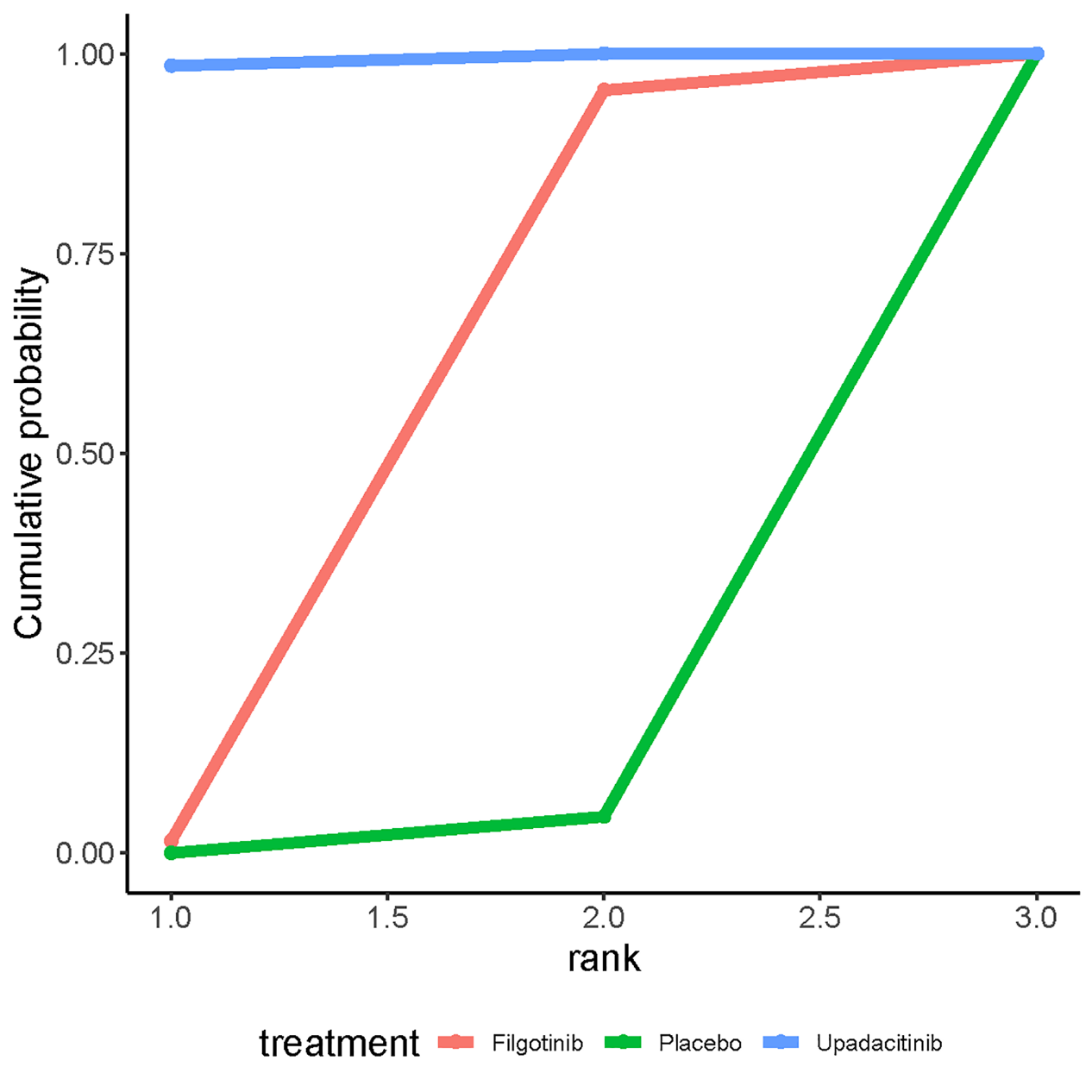


Figure S13 Line chart of endoscopic response in the Crohn's disease subgroup


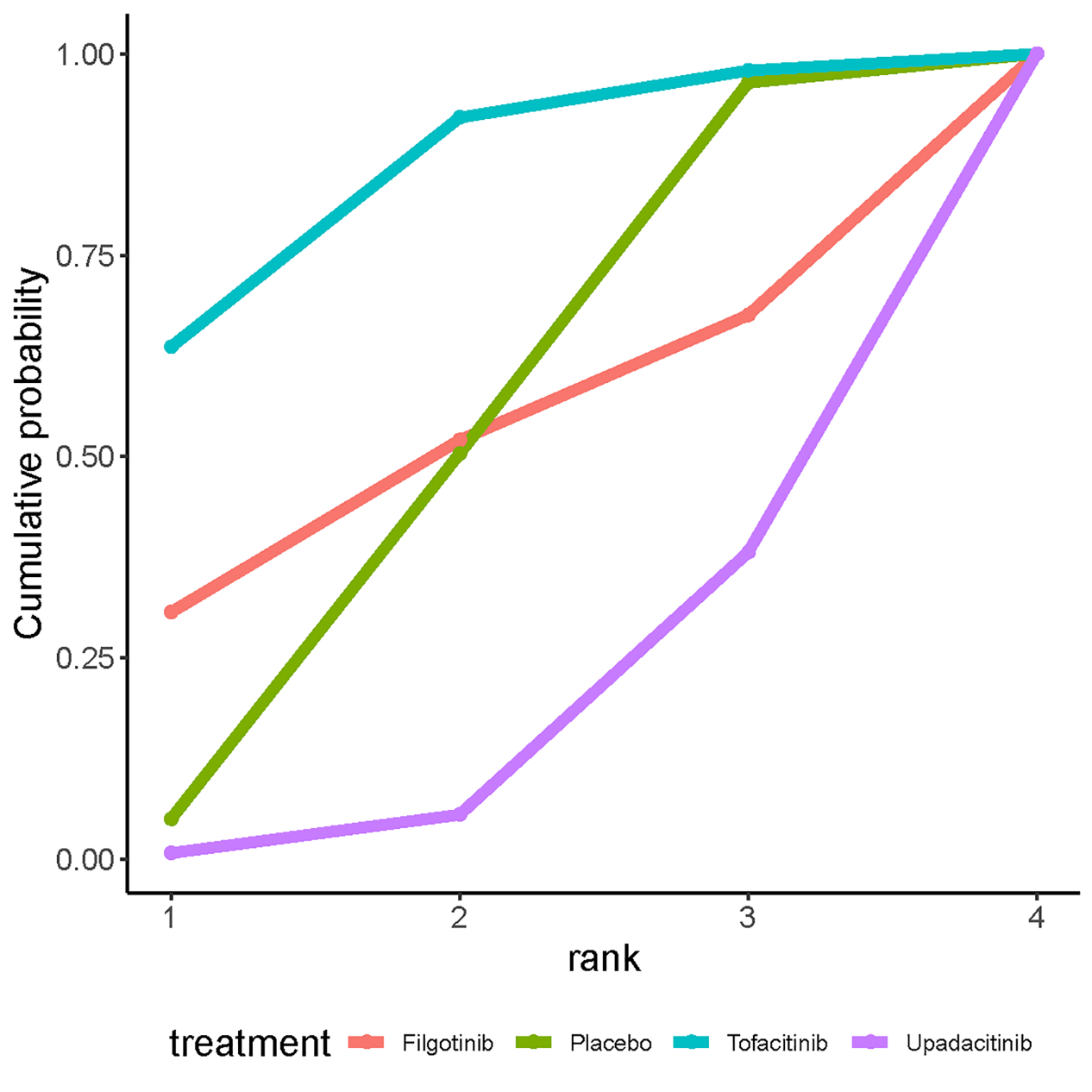


Figure S14 Line chart of adverse events in the Crohn's disease subgroup


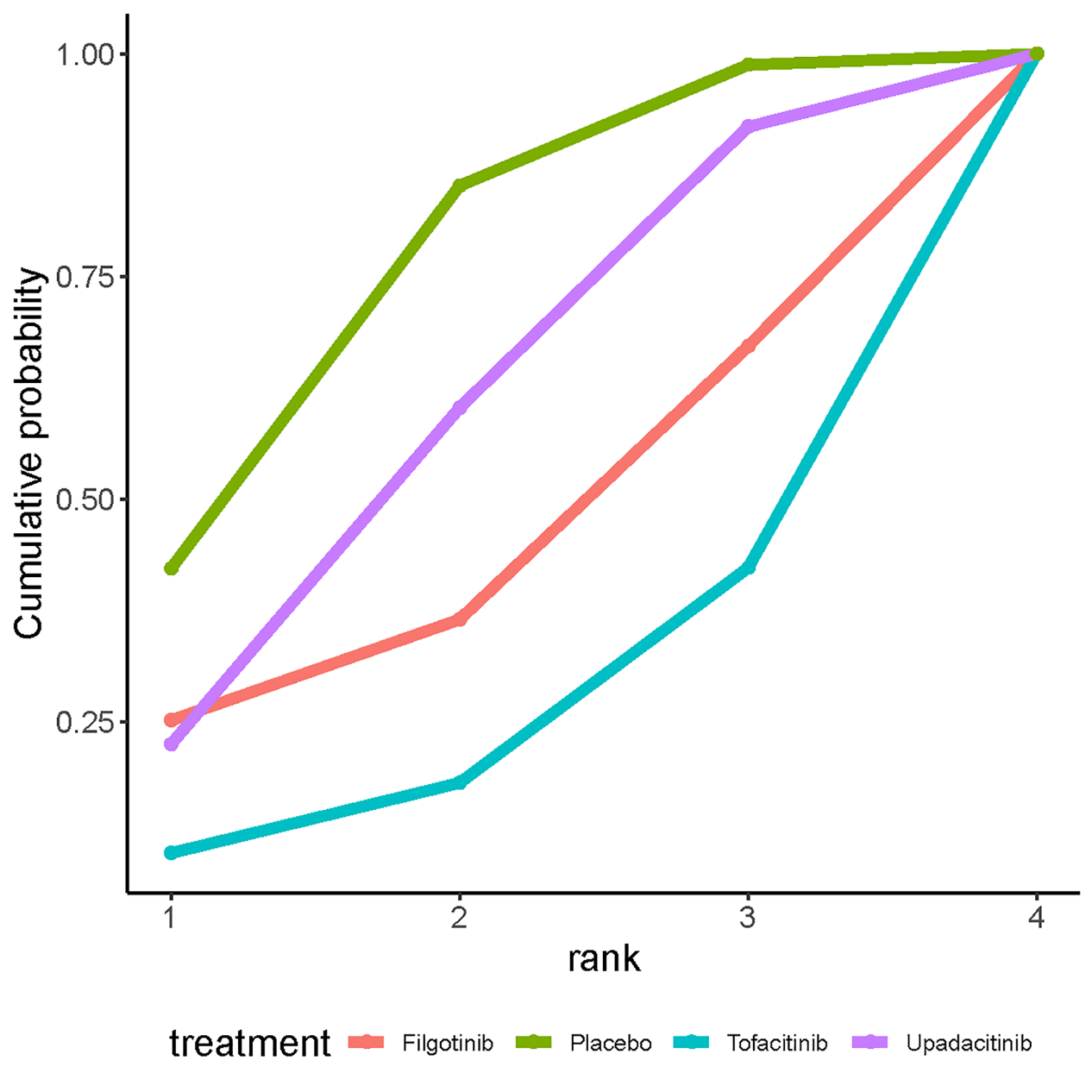


Figure S15 Line chart of serious adverse events in the Crohn's disease subgroup


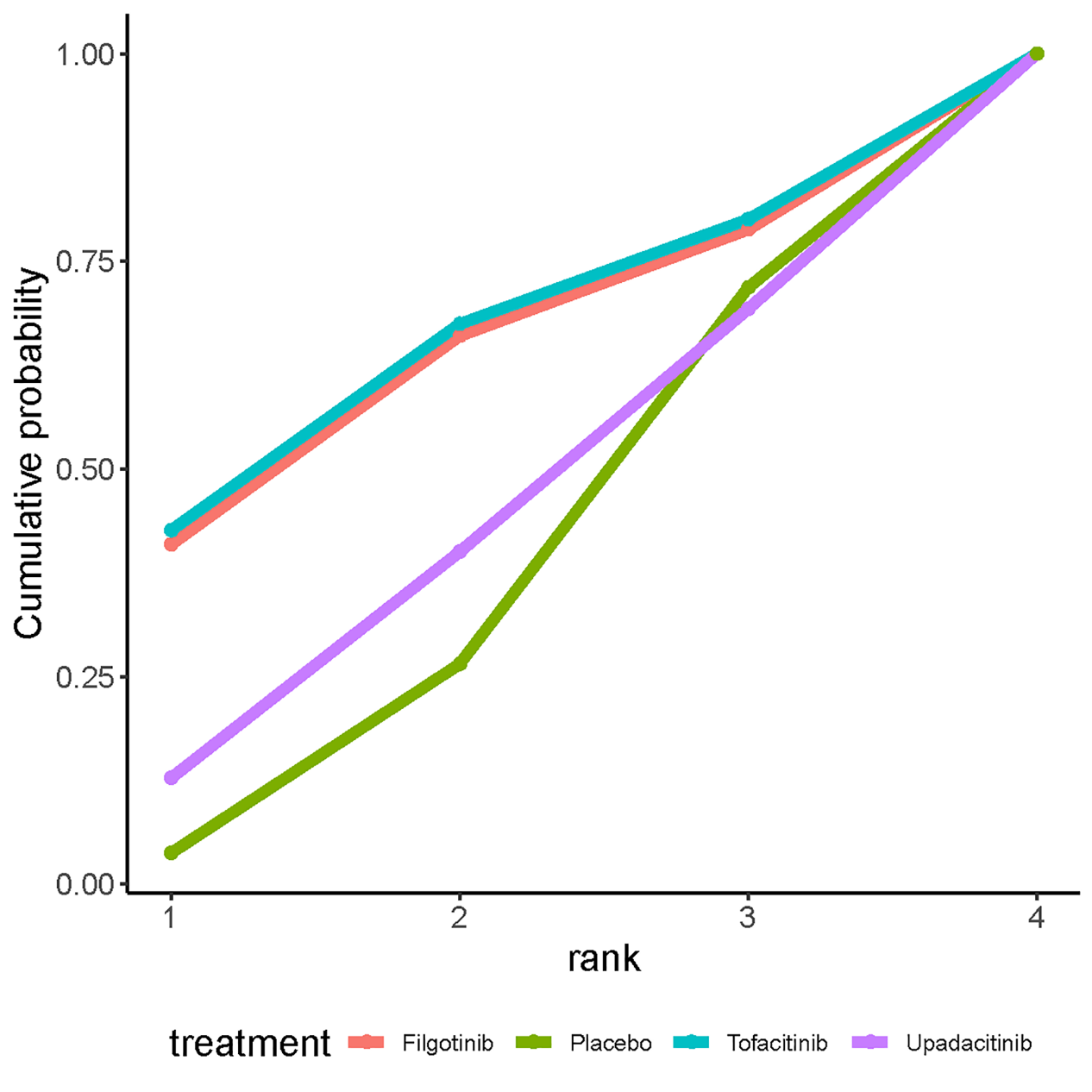


Figure S16 Line chart of adverse events leading to treatment discontinuation in the Crohn's disease subgroup


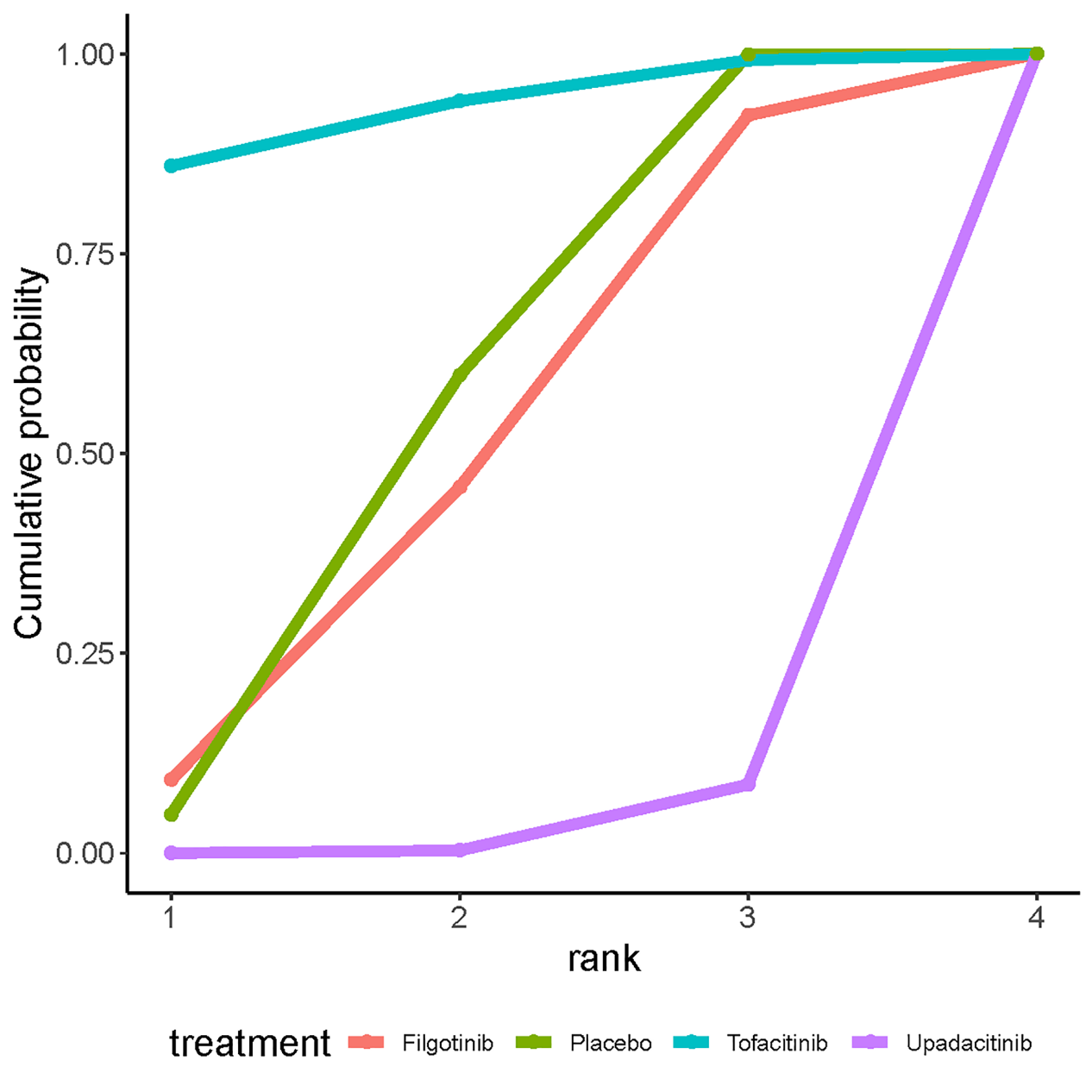


Figure S17 Line chart of infections in the Crohn's disease subgroup


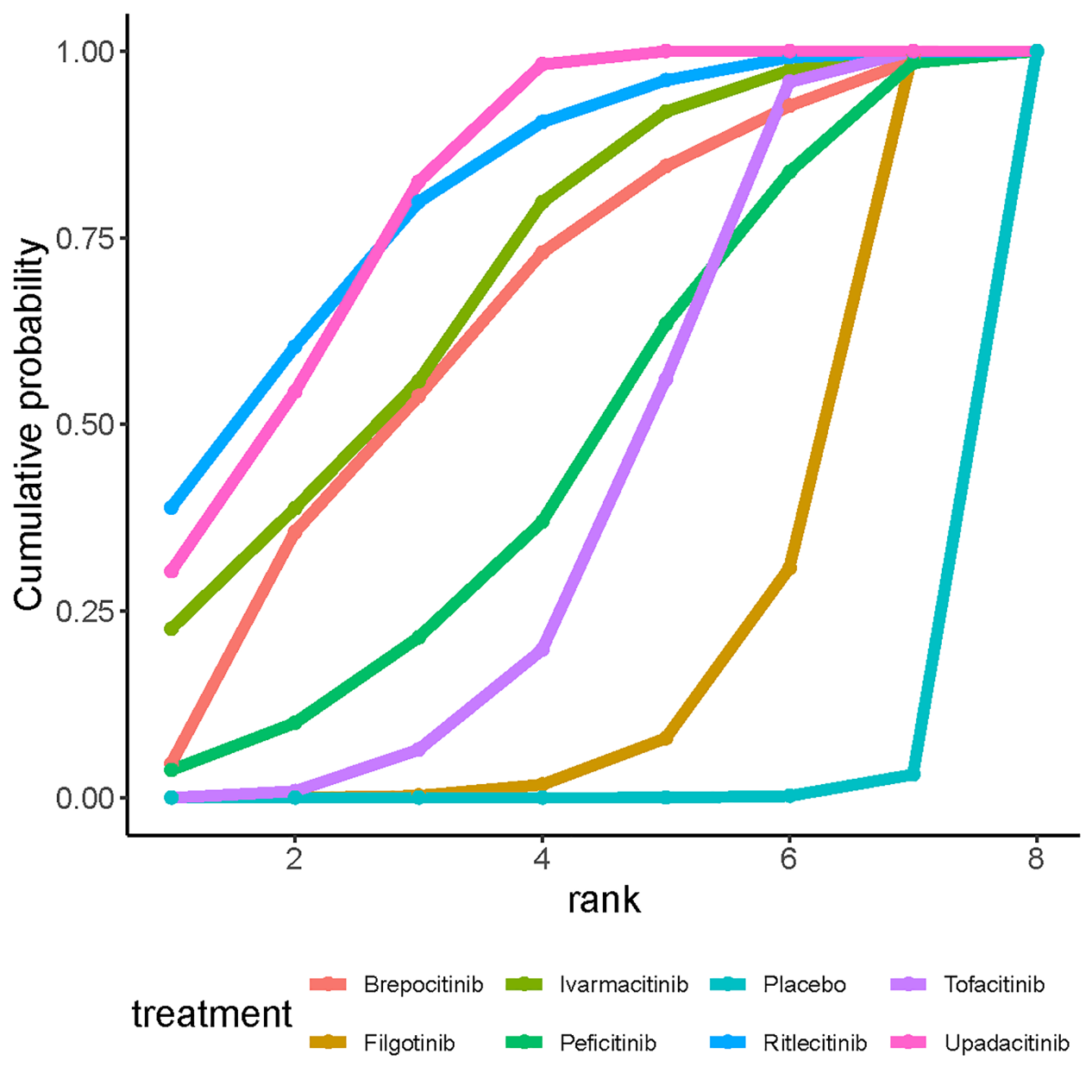


Figure S18 Line chart of clinical remission in the ulcerative colitis subgroup


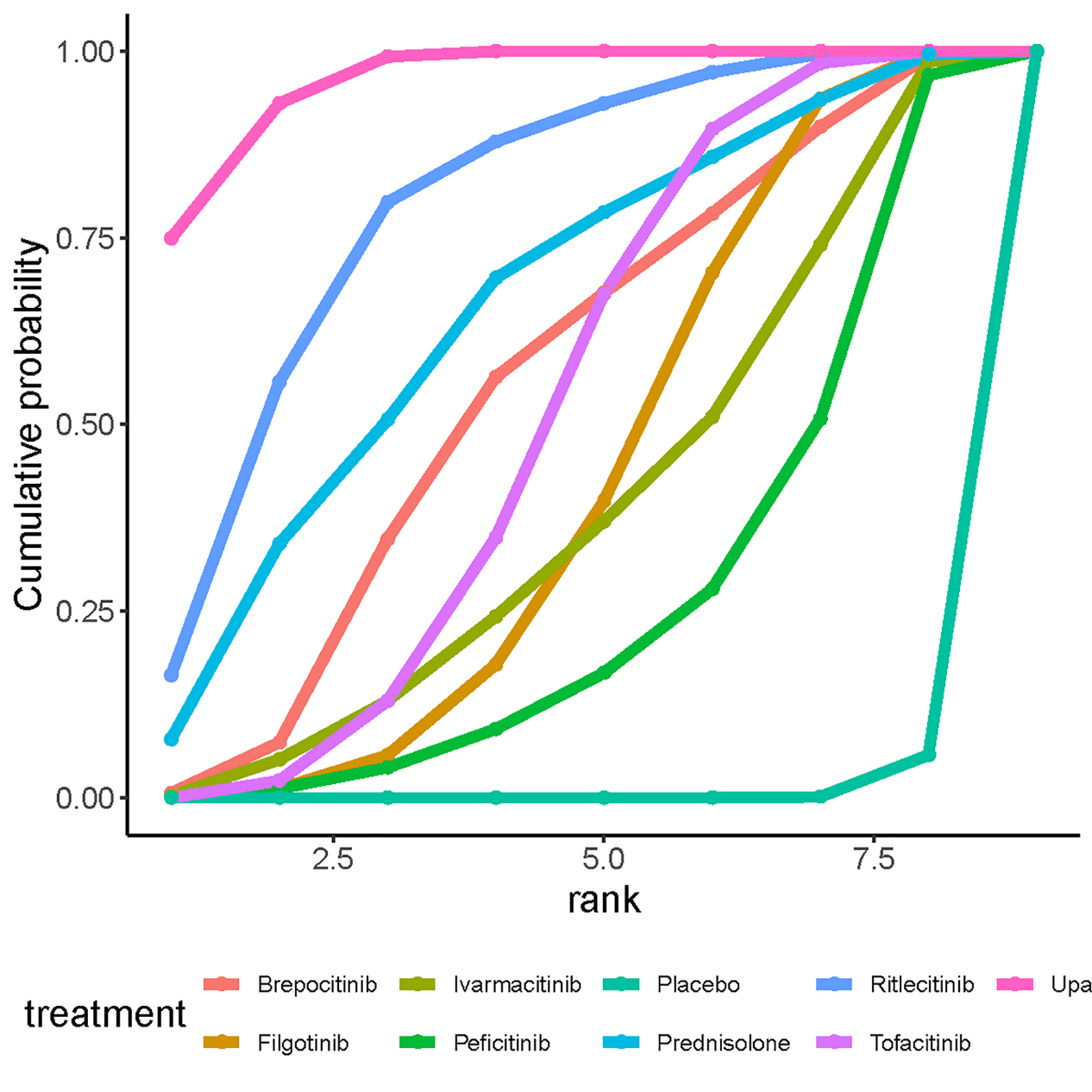


Figure S19 Line chart of clinical response in the ulcerative colitis subgroup


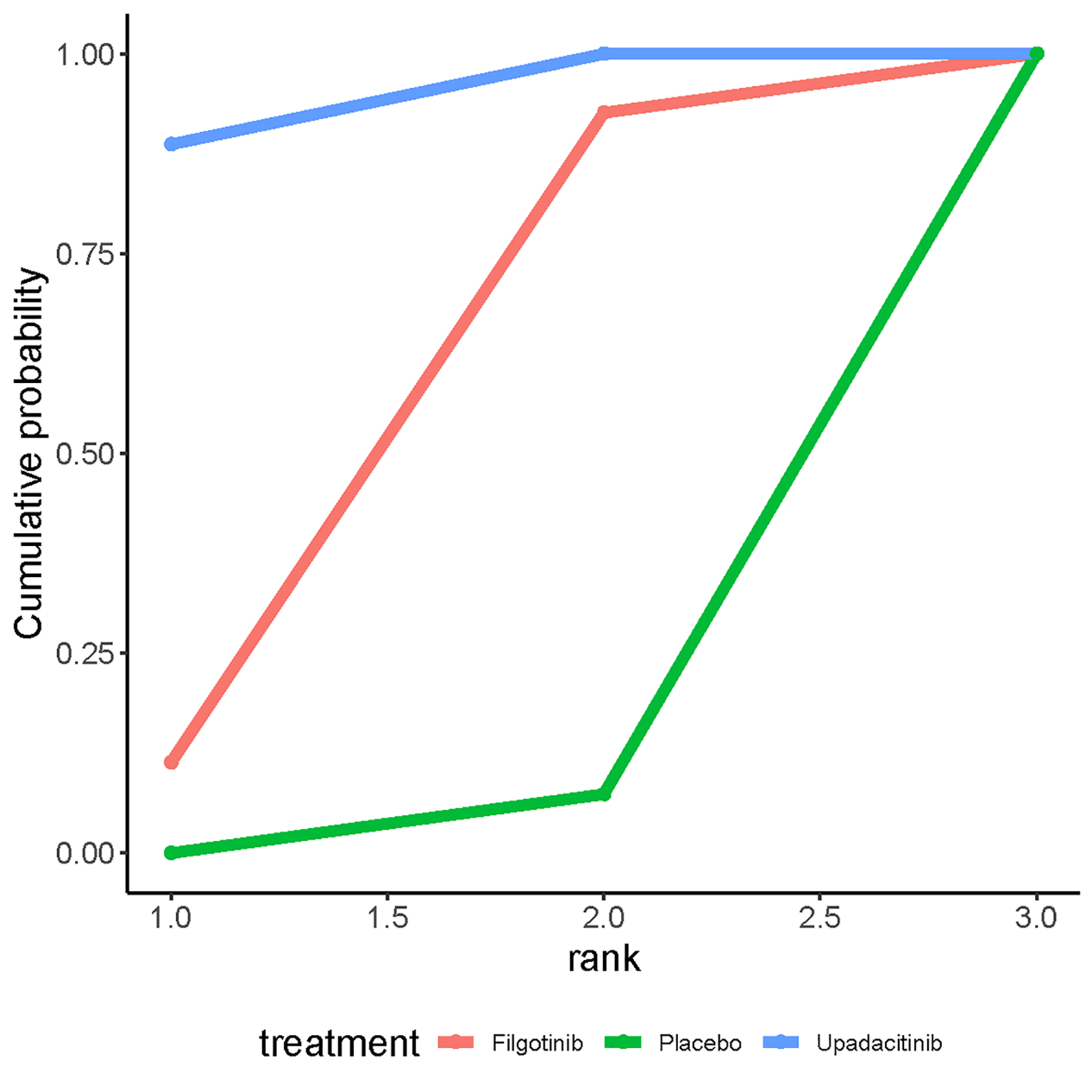


Figure S20 Line chart of endoscopic remission in the ulcerative colitis subgroup


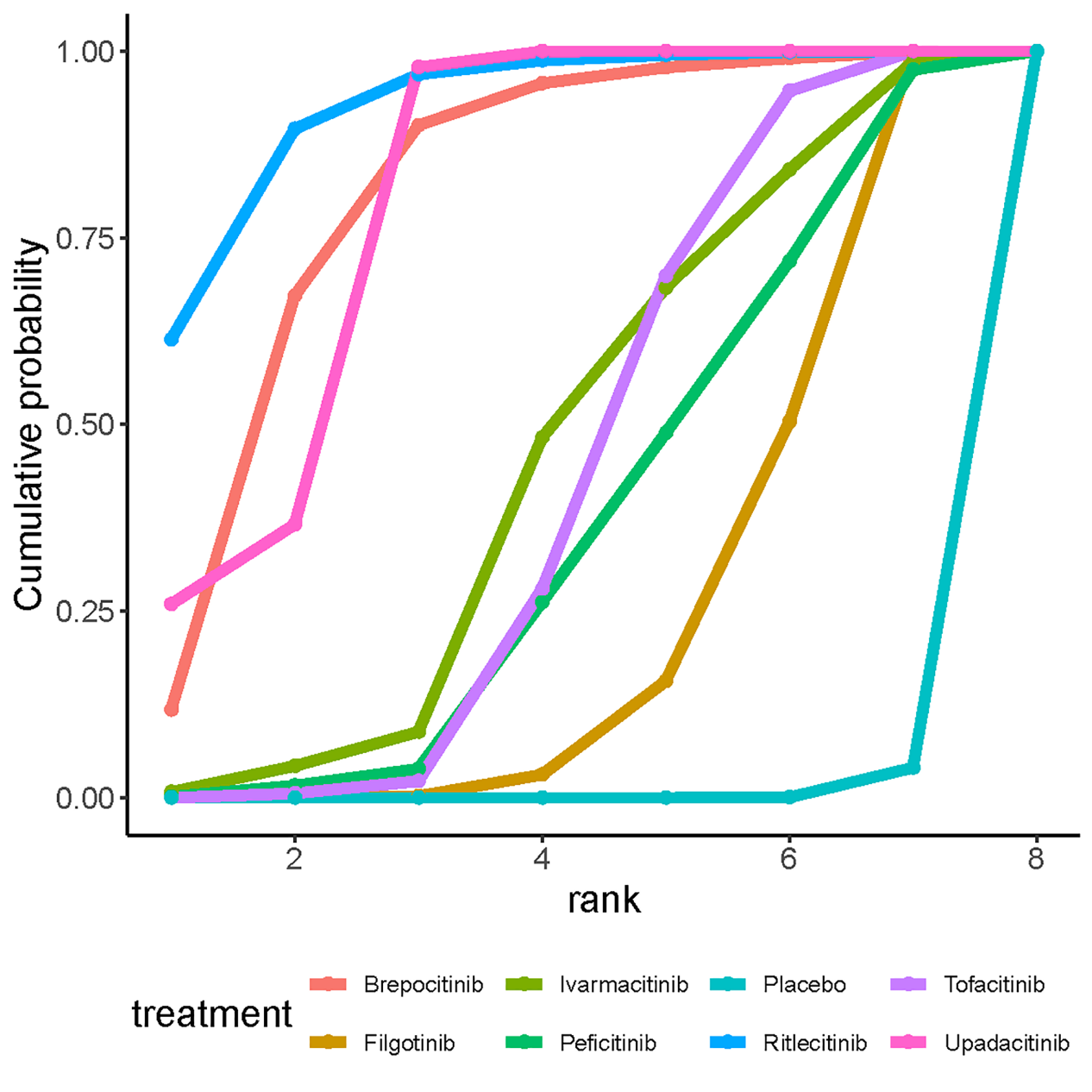


Figure S21 Line chart of endoscopic improvement in the ulcerative colitis subgroup


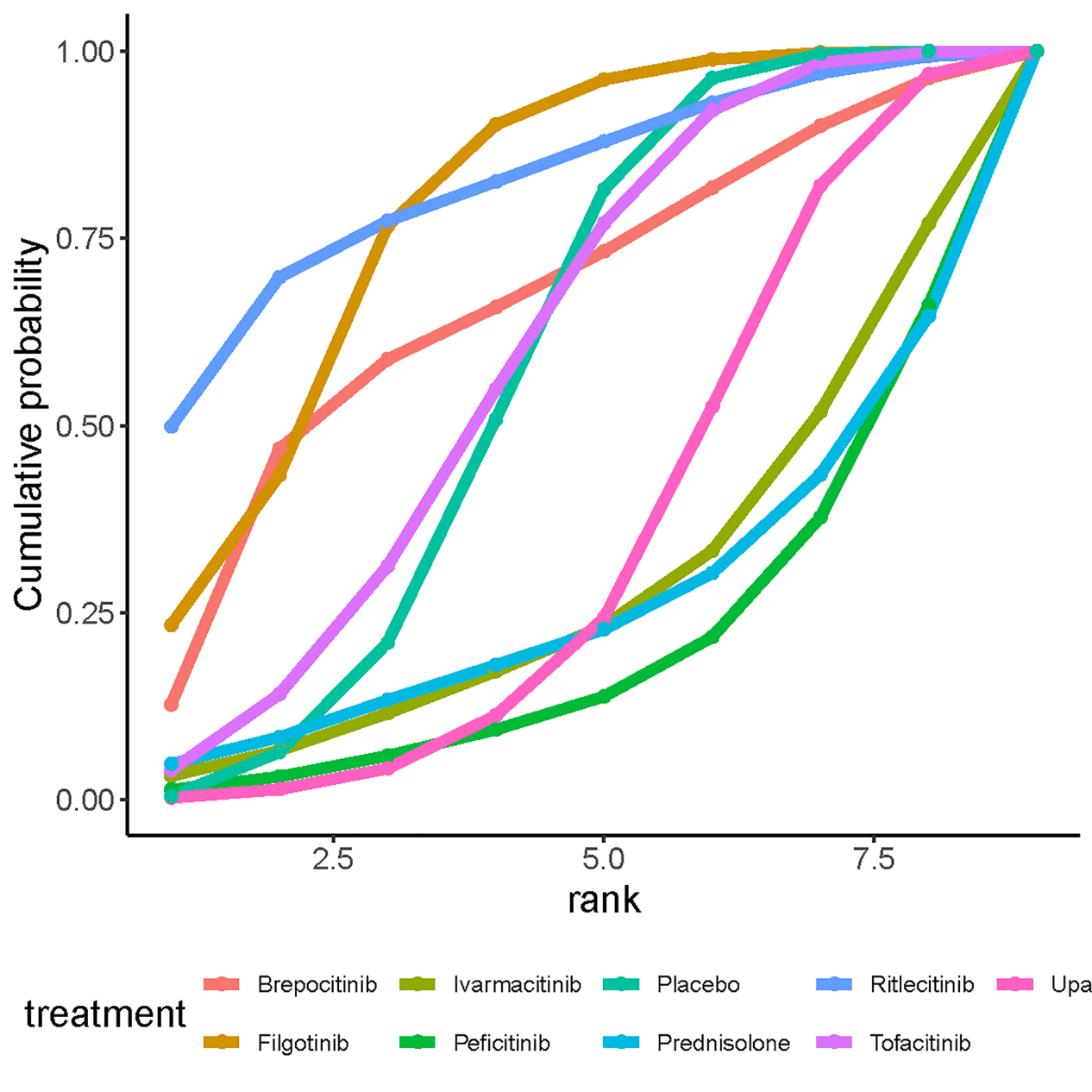


Figure S22 Line chart of adverse events in the ulcerative colitis subgroup


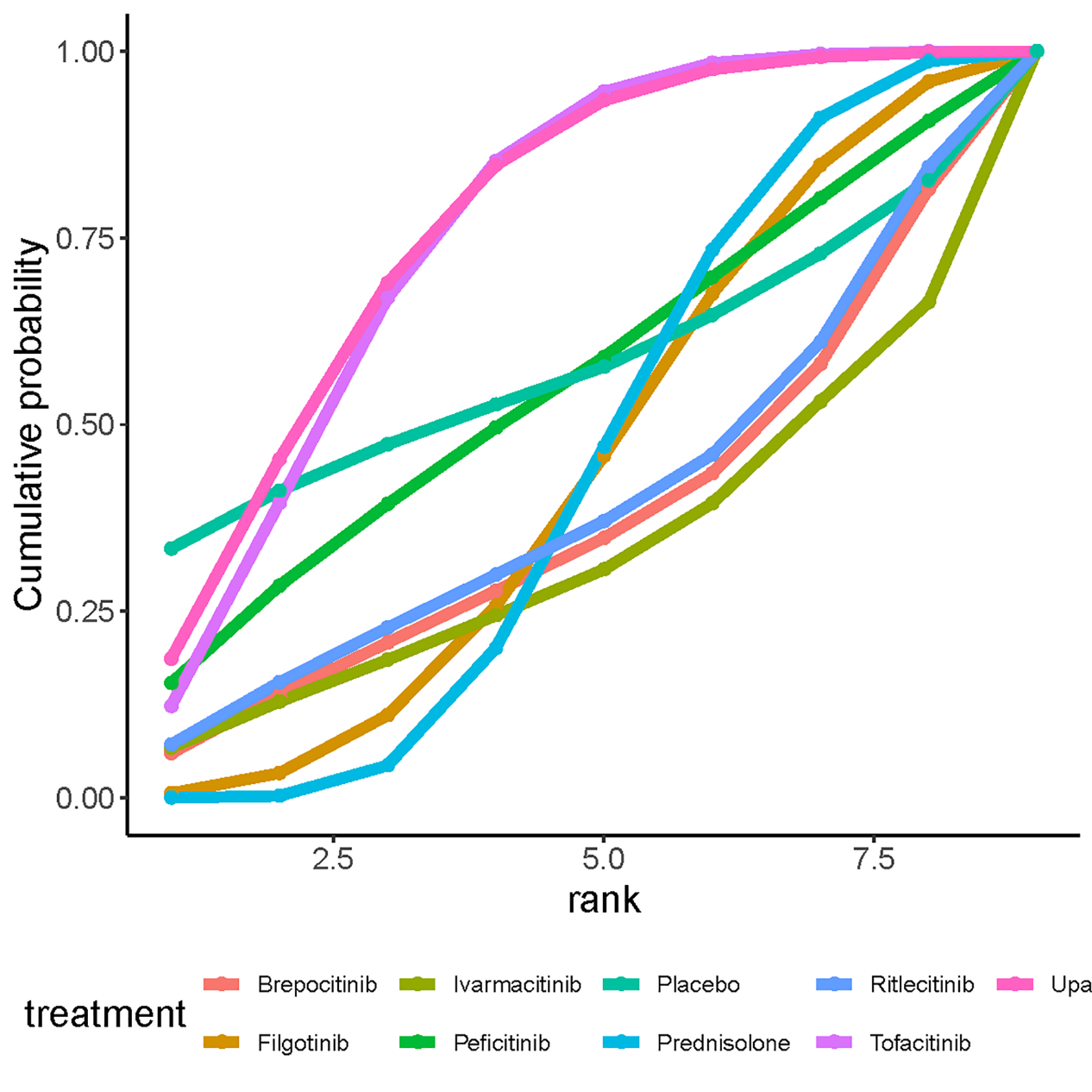


Figure S23 Line chart of serious adverse events in the ulcerative colitis subgroup


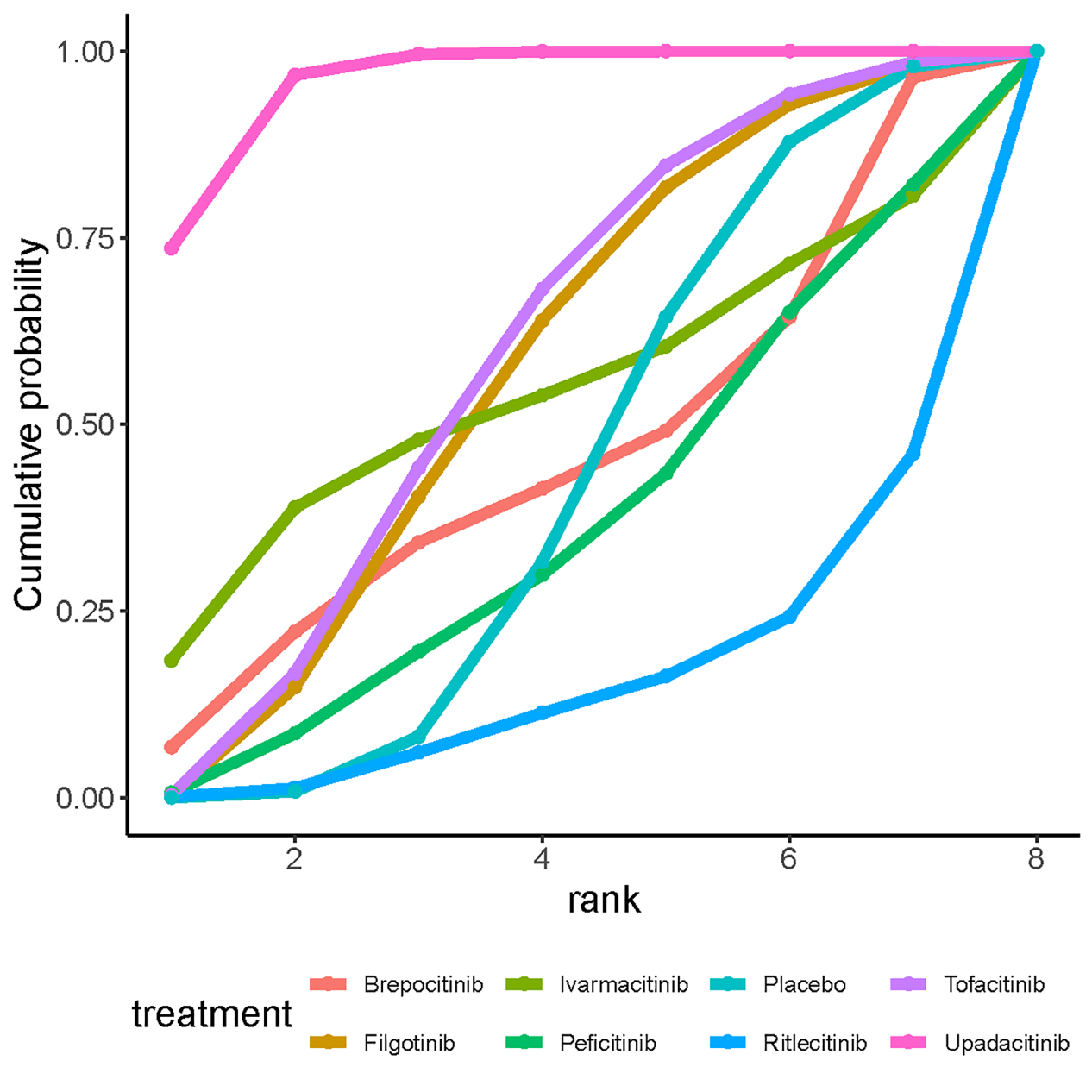


Figure S24 Line chart of adverse events leading to treatment discontinuation in the ulcerative colitis subgroup


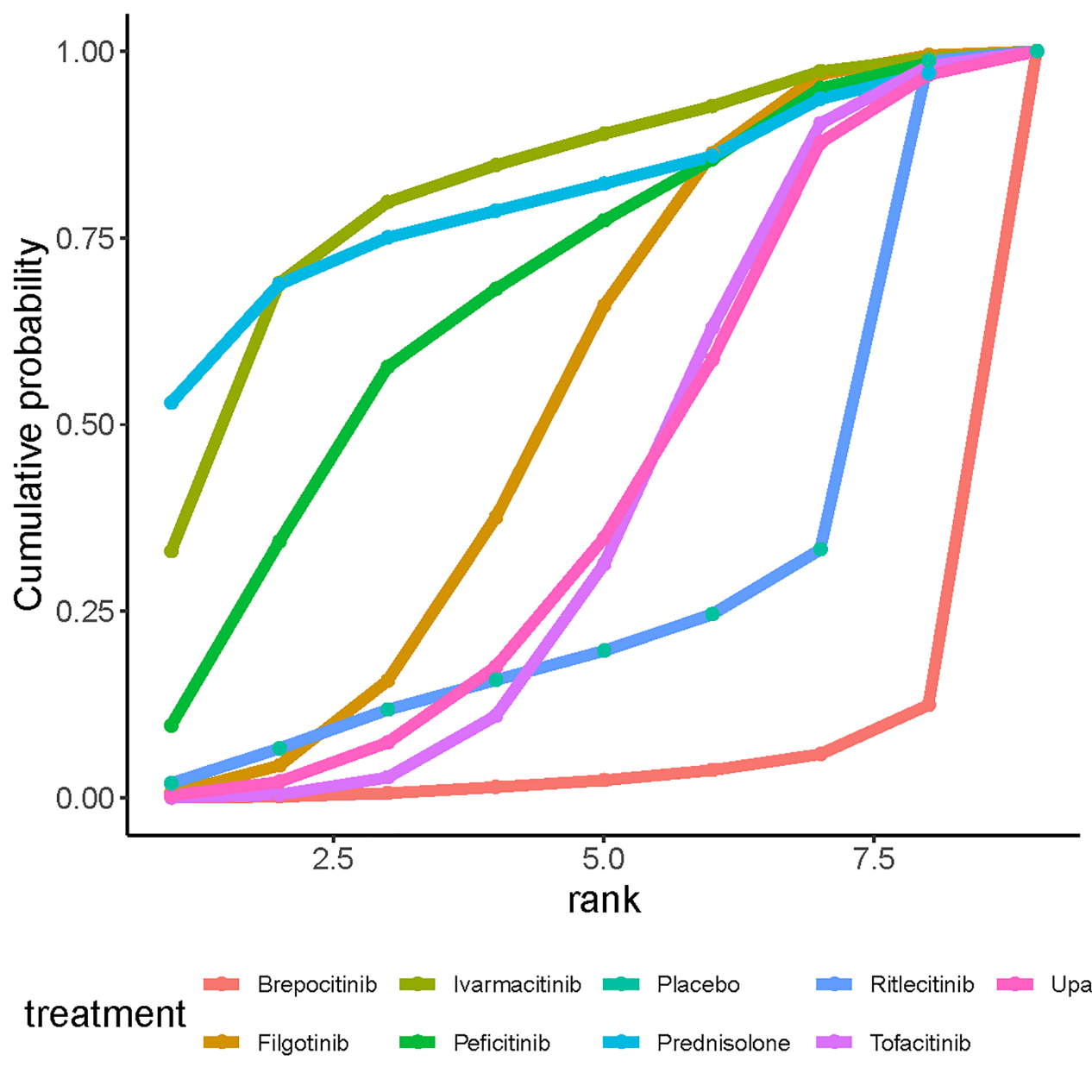


Figure S25 Line chart of infections in the ulcerative colitis subgroup


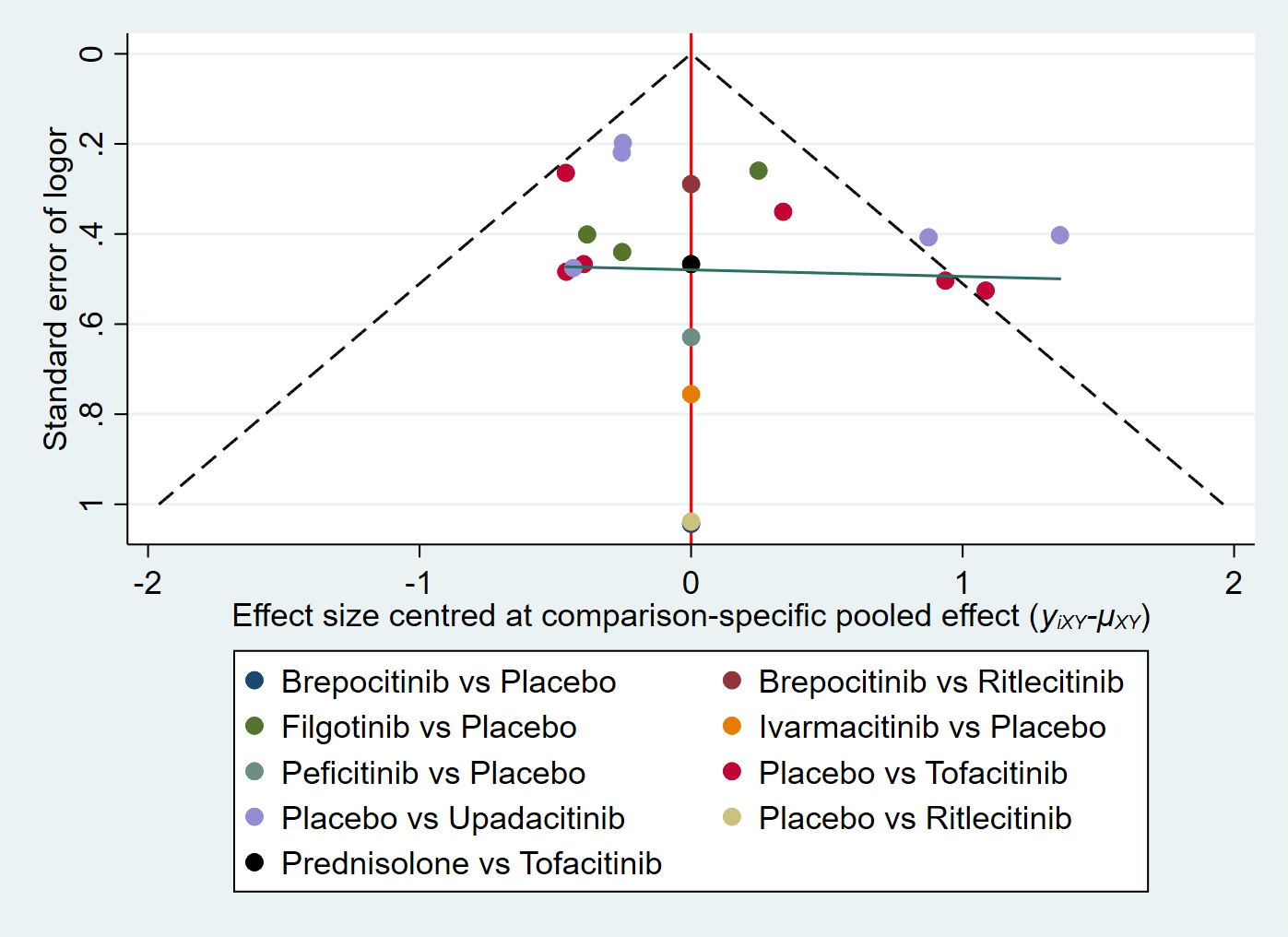


Figure S26 Funnel plot of clinical remission


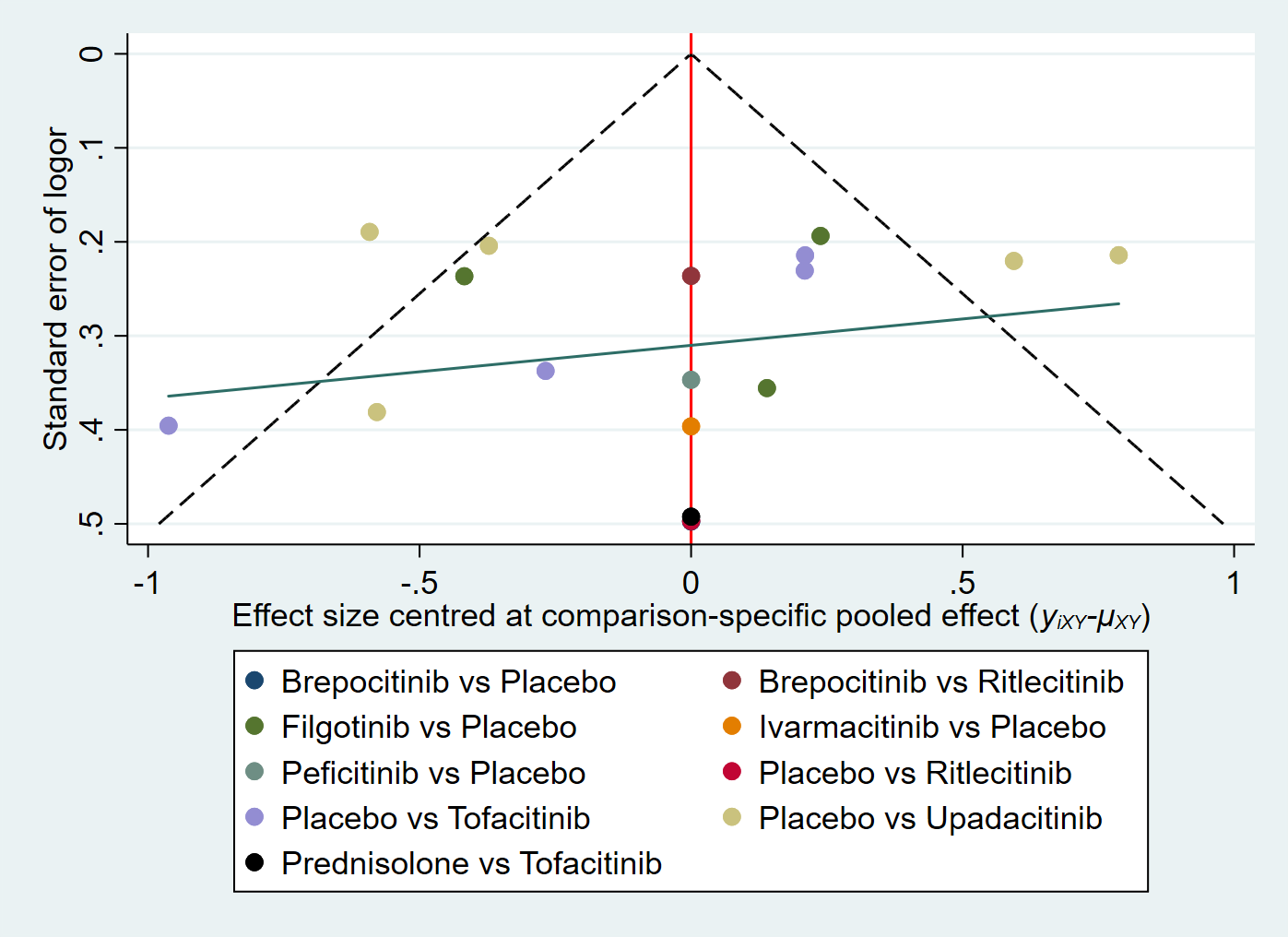


Figure S27 Funnel plot of Clinical response


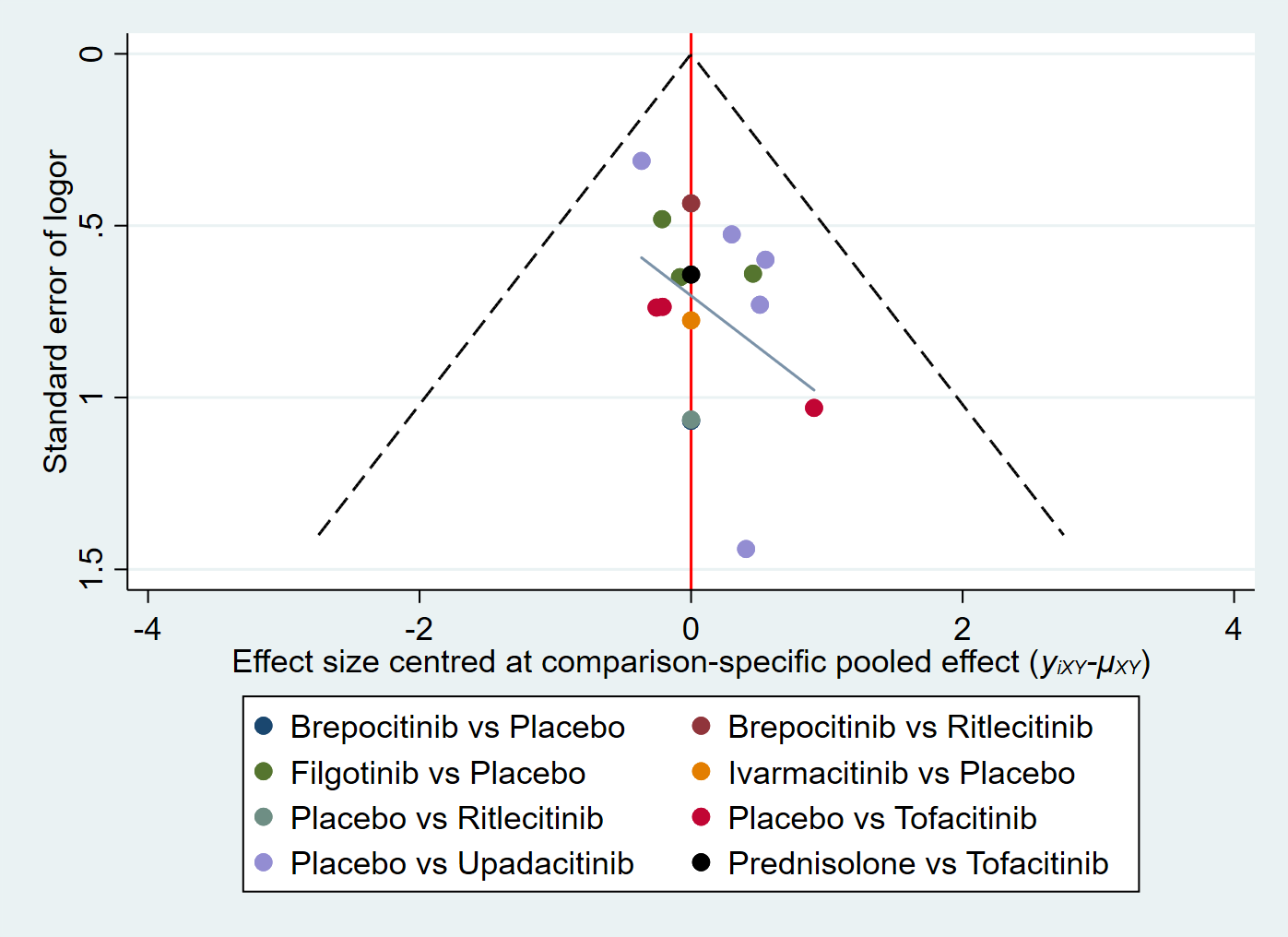


Figure S28 Funnel plot of Endoscopic remission


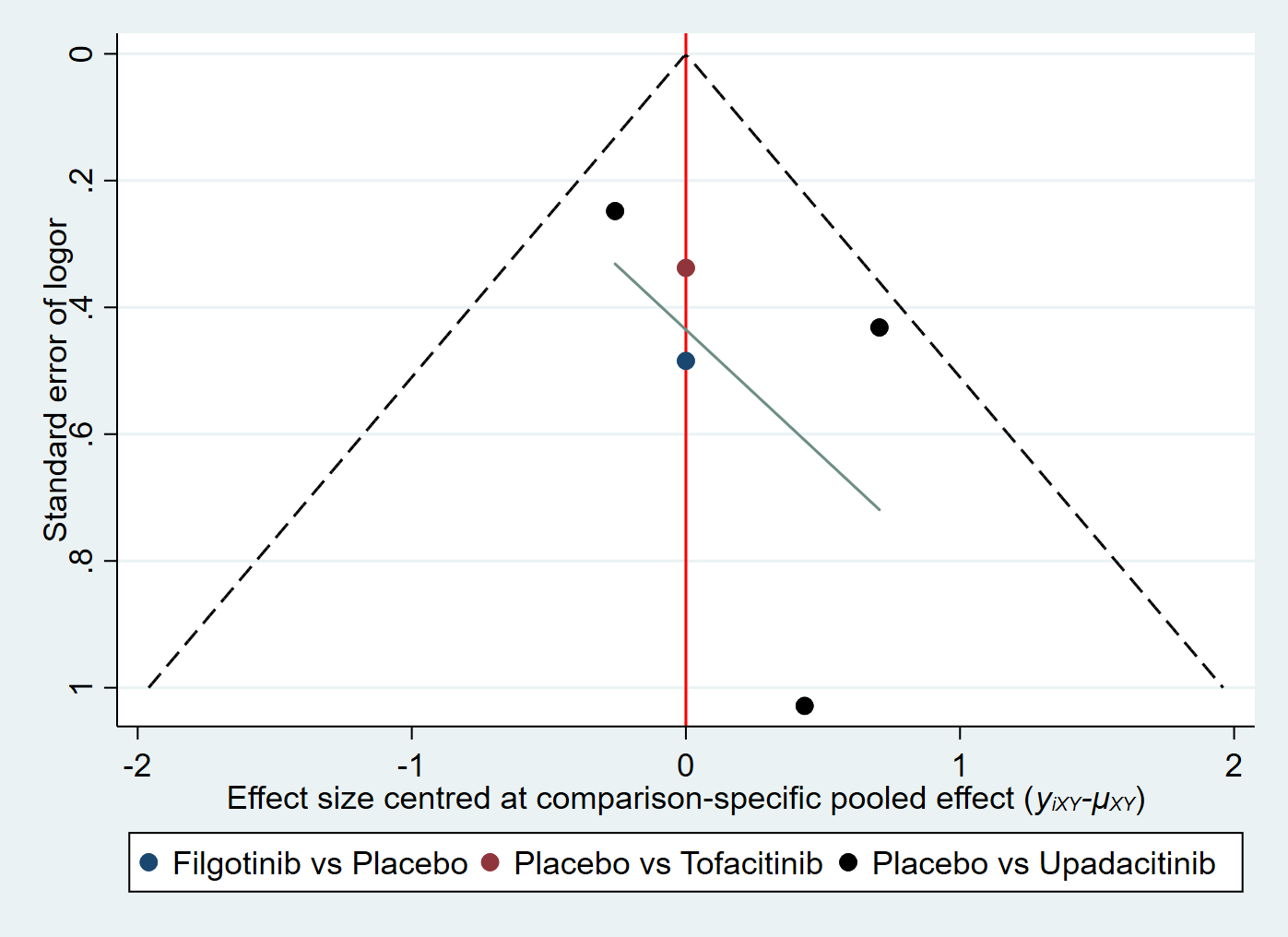
 Figure S29 Funnel plot of Endoscopic response


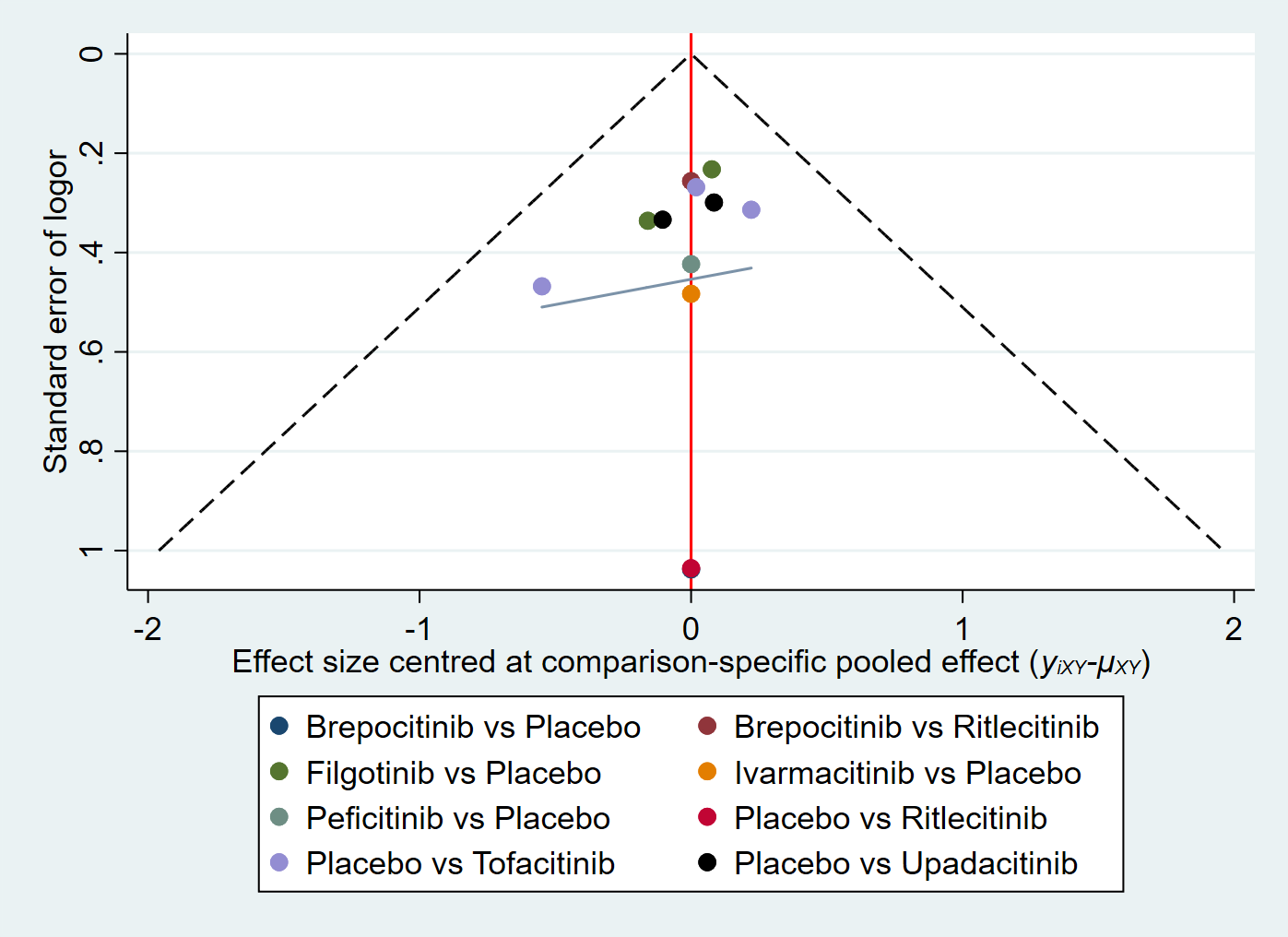
 Figure S30 Funnel plot of Endoscopic improvement


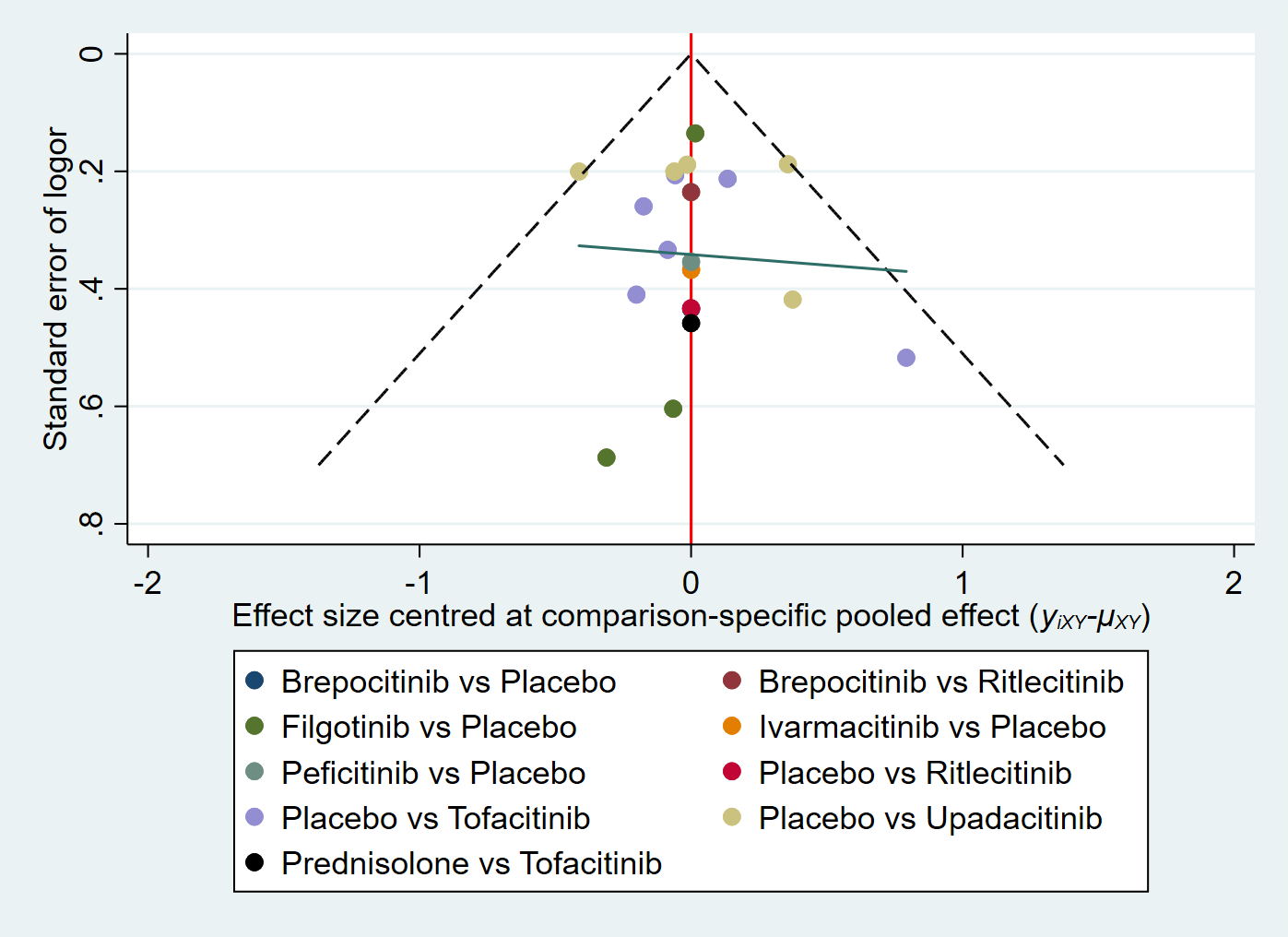
 Figure S31 Funnel plot of Adverse event


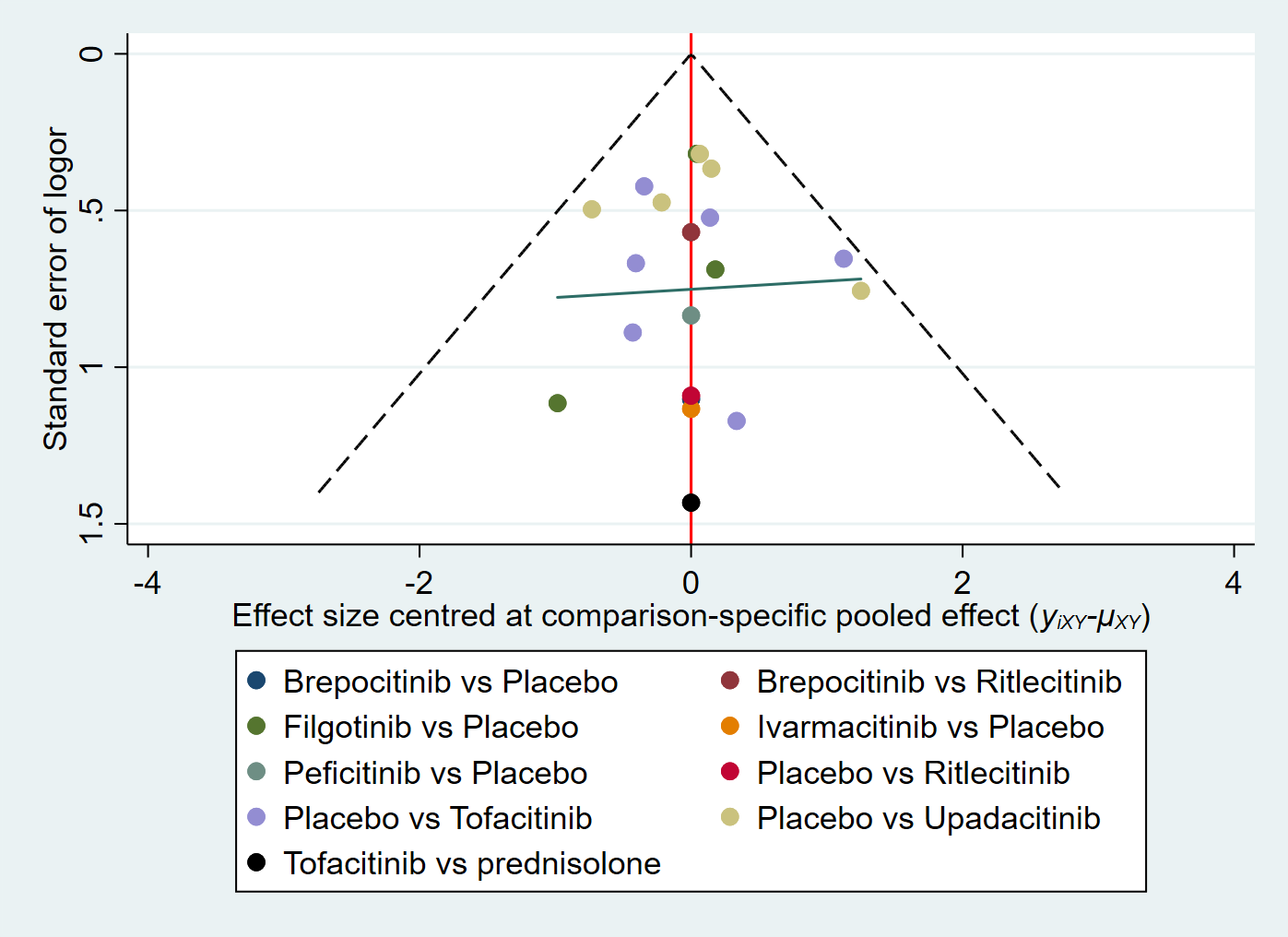


Figure S32 Funnel plot of serious adverse events
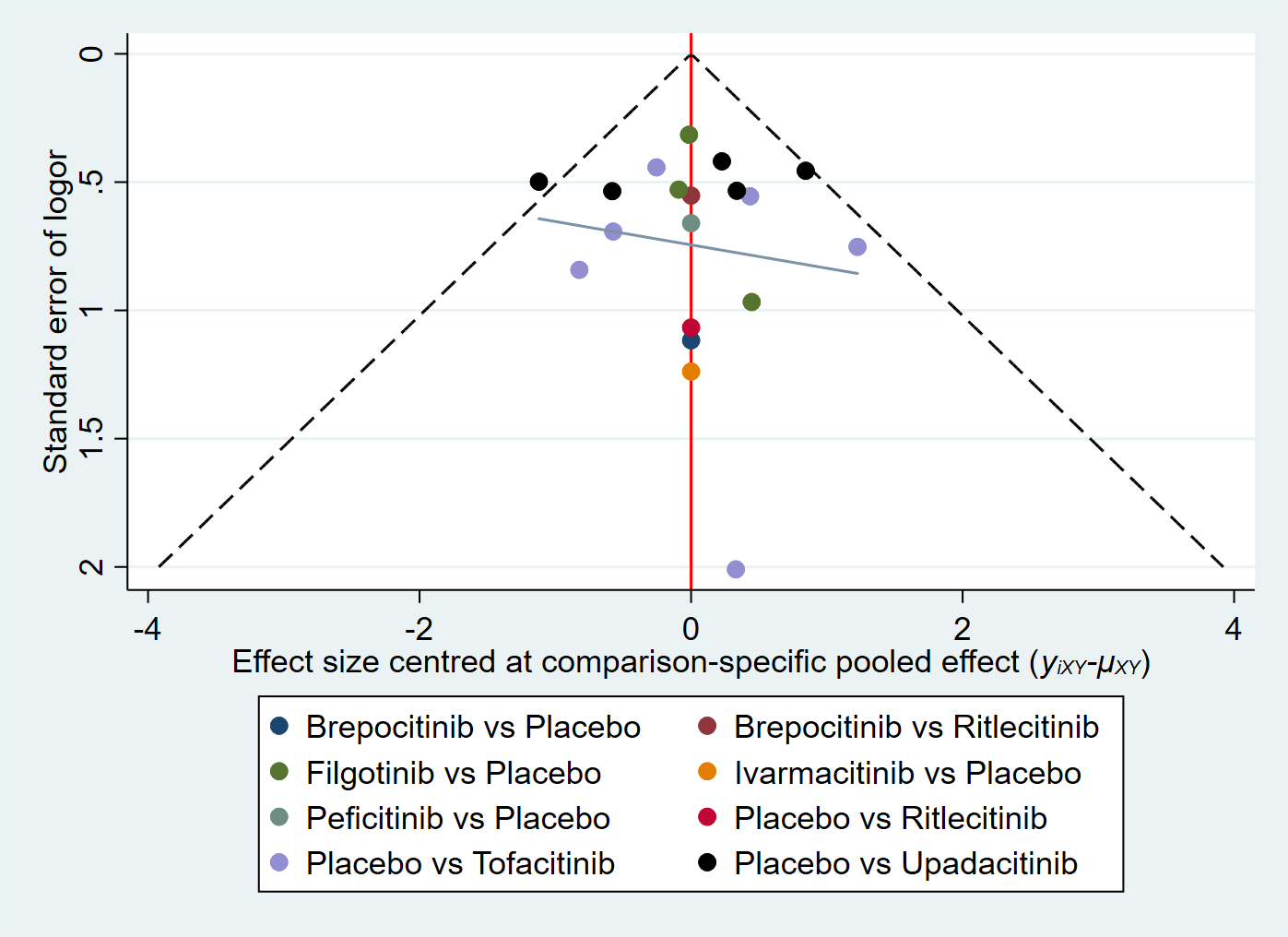


Figure S33 Funnel plot of adverse events leading to treatment discontinuation


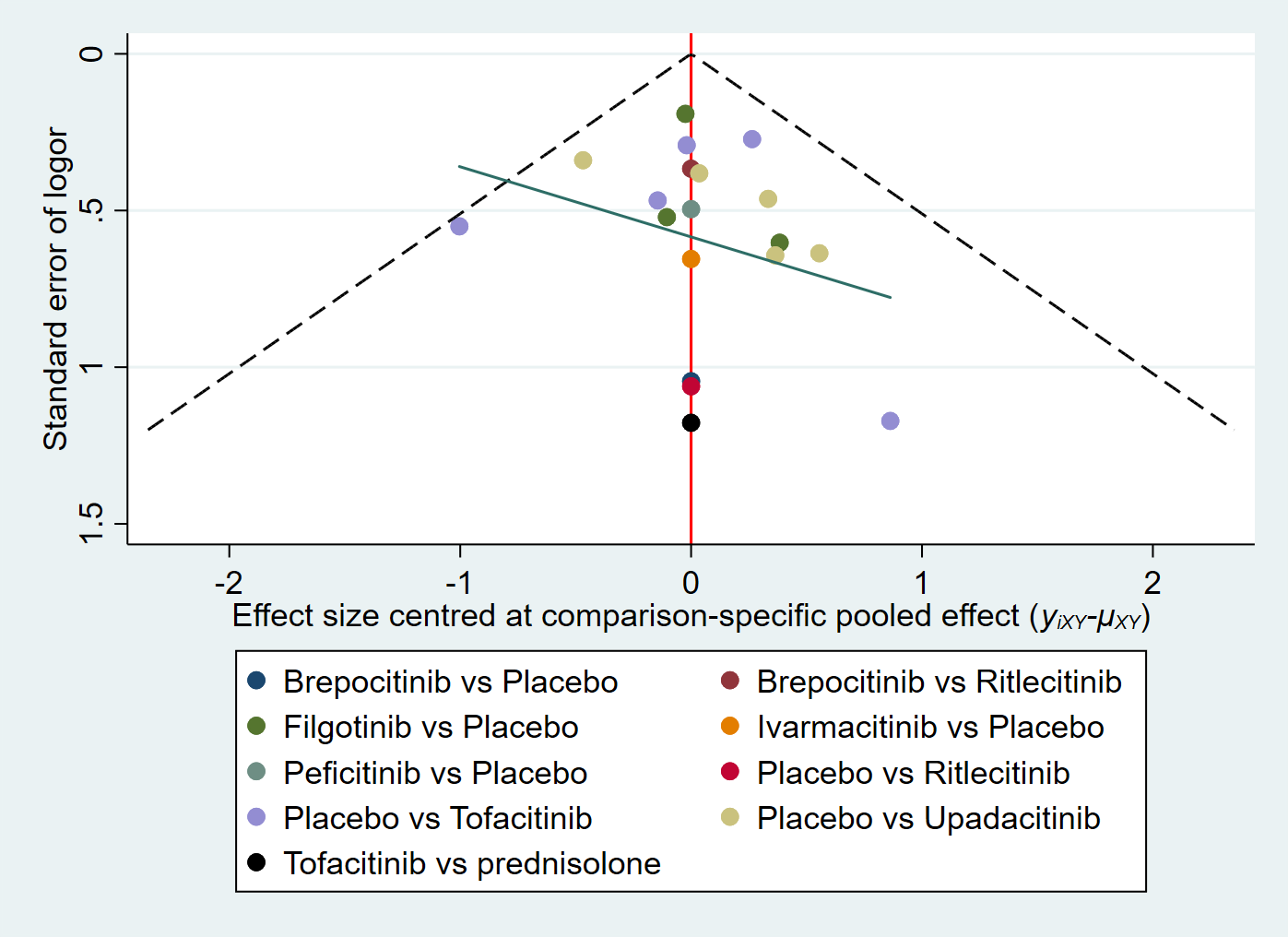


Figure S34 Funnel plot of infections

Table S1 League table for clinical remission in the Crohn's disease subgroup

| OR 95%CI | | | |
| --- | --- | --- | --- |
| Filgotinib |  |  |  |
| 3.08 (1.43, 7.11) * | Placebo |  |  |
| 2.69 (1.1, 6.91) * | 0.87 (0.55, 1.36) | Tofacitinib |  |
| 1.3 (0.58, 3.14) | 0.42 (0.32, 0.56) * | 0.49 (0.29, 0.82) * | Upadacitinib |

* means P<0.05

Table S2 League table for clinical response in the Crohn's disease subgroup

| OR 95%CI | | | |
| --- | --- | --- | --- |
| Filgotinib |  |  |  |
| 2.13 (1.06, 4.36) * | Placebo |  |  |
| 2.24 (0.79, 6.47) | 1.06 (0.48, 2.29) | Tofacitinib |  |
| 0.89 (0.42, 1.91) | 0.42 (0.32, 0.54) * | 0.4 (0.18, 0.9) * | Upadacitinib |

*means P<0.05

Table S3 League table for endoscopic remission in the Crohn's disease subgroup

| OR 95%CI | | |
| --- | --- | --- |
| Filgotinib |  |  |
| 2.45 (0.75, 11.5) | Placebo |  |
| 0.36 (0.1, 1.82) | 0.15 (0.09, 0.24)* | Upadacitinib |

*means P<0.05

Table S4 League table for endoscopic response in the Crohn's disease subgroup

| OR 95%CI | | |
| --- | --- | --- |
| Filgotinib |  |  |
| 2.2 (0.89, 6.38) | Placebo |  |
| 0.28 (0.1, 0.88)* | 0.13 (0.08, 0.19)* | Upadacitinib |

*means P<0.05

Table S5 League table for adverse events in the Crohn's disease subgroup

| OR 95%CI | | | |
| --- | --- | --- | --- |
| Filgotinib |  |  |  |
| 0.98 (0.38, 2.34) | Placebo |  |  |
| 1.28 (0.45, 3.39) | 1.3 (0.85, 2.01) | Tofacitinib |  |
| 0.81 (0.3, 2.01) | 0.83 (0.64, 1.07) | 0.64 (0.38, 1.04) | Upadacitinib |

*means P<0.05

Table S6 League table for serious adverse events in the Crohn's disease subgroup

| OR 95%CI | | | |
| --- | --- | --- | --- |
| Filgotinib |  |  |  |
| 1.39 (0.47, 5.19) | Placebo |  |  |
| 0.75 (0.14, 4.02) | 0.54 (0.15, 1.57) | Tofacitinib |  |
| 1.24 (0.38, 4.96) | 0.9 (0.57, 1.38) | 1.66 (0.52, 6.49) | Upadacitinib |

*means P<0.05

Table S7 League table for adverse events leading to treatment discontinuation in the Crohn's disease subgroup

| OR 95%CI | | | |
| --- | --- | --- | --- |
| Filgotinib |  |  |  |
| 0.77 (0.32, 2.06) | Placebo |  |  |
| 1.02 (0.28, 3.81) | 1.32 (0.51, 3.2) | Tofacitinib |  |
| 0.8 (0.28, 2.42) | 1.04 (0.6, 1.73) | 0.79 (0.28, 2.32) | Upadacitinib |

*means P<0.05

Table S8 League table for infections in the Crohn's disease subgroup

| OR 95%CI | | | |
| --- | --- | --- | --- |
| Filgotinib |  |  |  |
| 1.06 (0.5, 2.35) | Placebo |  |  |
| 2.3 (0.59, 8.97) | 2.16 (0.7, 6.62) | Tofacitinib |  |
| 0.5 (0.19, 1.31) | 0.47 (0.26, 0.81)* | 0.22 (0.06, 0.76)* | Upadacitinib |

Table S9 League table for clinical remission in the ulcerative colitis subgroup

| OR 95%CI | | | | | | | |
| --- | --- | --- | --- | --- | --- | --- | --- |
| Brepocitinib |  |  |  |  |  |  |  |
| 3.83 (0.61, 93.33) | Filgotinib |  |  |  |  |  |  |
| 1.01 (0.07, 29.64) | 0.26 (0.04, 1.09) | Ivarmacitinib |  |  |  |  |  |
| 2.11 (0.2, 58.4) | 0.55 (0.11, 1.87) | 2.09 (0.28, 20.06) | Peficitinib |  |  |  |  |
| 7.15 (1.22, 170.78)* | 1.87 (1.23, 2.93)* | 7.09 (1.85, 51.19)* | 3.4 (1.1, 15.68)* | Placebo |  |  |  |
| 0.71 (0.4, 1.25) | 0.19 (0.01, 1.14) | 0.71 (0.02, 9.99) | 0.34 (0.01, 3.47) | 0.1 (0, 0.57)* | Ritlecitinib |  |  |
| 2.42 (0.39, 59.47) | 0.63 (0.35, 1.16) | 2.4 (0.58, 18.02) | 1.15 (0.34, 5.55) | 0.34 (0.22, 0.5)* | 3.41 (0.55, 83.86) | Tofacitinib |  |
| 0.72 (0.11, 17.67) | 0.19 (0.09, 0.38)* | 0.72 (0.16, 5.52) | 0.34 (0.1, 1.72) | 0.1 (0.06, 0.17)* | 1.01 (0.16, 25.07) | 0.3 (0.14, 0.6)* | Upadacitinib |

*means P<0.05

Table S10 NMA subgroup analysis: SUCRA ranking for ulcerative colitis

| Treatment | Clinical remission (%) | Clinical response (%) | Endoscopic remission (%) | Endoscopic improvement (%) | Adverse events (%) | Serious adverse events (%) | Adverse events leading to treatment discontinuation (%) | Infections (%) |
| --- | --- | --- | --- | --- | --- | --- | --- | --- |
| Brepocitinib | 63.3 | 54.3 | - | 80.2 | 65.7 | 35.8 | 44.9 | 3.3 |
| Filgotinib | 20.1 | 41.0 | 52 | 24.2 | 78.5 | 41.8 | 56 | 50.8 |
| Ivarmacitinib | 69.4 | 37.9 | - | 44.7 | 28 | 31.5 | 53.1 | 80.5 |
| Peficitinib | 45.4 | 25.8 | - | 35.7 | 19.9 | 54 | 35.6 | 65.8 |
| Ritlecitinib | 80.7 | 78.7 | - | 92.3 | 82.1 | 38 | 15.1 | 26.5 |
| Tofacitinib | 39.7 | 50.7 | - | 42.2 | 58.9 | 74.6 | 58.1 | 37.1 |
| Upadacitinib | 80.1 | 95.9 | 94.3 | 80.1 | 34.1 | 76 | 95.7 | 38.2 |
| Placebo | 0.5 | 0.7 | 3.7 | 0.6 | 57 | 41.8 | 41.5 | 68.5 |
| Prednisolone | - | 64.9 | - | - | 25.7 | 56.6 | - | 79.3 |

Table S11 League table for clinical response in the ulcerative colitis subgroup

| OR 95%CI | | | | | | | | |
| --- | --- | --- | --- | --- | --- | --- | --- | --- |
| Brepocitinib |  |  |  |  |  |  |  |  |
| 1.29 (0.48, 3.93) | Filgotinib |  |  |  |  |  |  |  |
| 1.41 (0.41, 5.23) | 1.09 (0.46, 2.46) | Ivarmacitinib |  |  |  |  |  |  |
| 1.73 (0.53, 6.17) | 1.34 (0.63, 2.8) | 1.23 (0.44, 3.56) | Peficitinib |  |  |  |  |  |
| 3.27 (1.27, 9.6)* | 2.53 (1.91, 3.38)* | 2.32 (1.09, 5.27)* | 1.9 (0.96, 3.83) | Placebo |  |  |  |  |
| 0.86 (0.21, 3.7) | 0.66 (0.23, 1.89) | 0.61 (0.17, 2.21) | 0.5 (0.14, 1.69) | 0.26 (0.09, 0.72)* | Prednisolone |  |  |  |
| 0.68 (0.43, 1.09) | 0.53 (0.17, 1.43) | 0.48 (0.13, 1.69) | 0.4 (0.11, 1.29) | 0.21 (0.07, 0.54)* | 0.8 (0.19, 3.2) | Ritlecitinib |  |  |
| 1.15 (0.43, 3.51) | 0.89 (0.6, 1.33) | 0.82 (0.36, 1.95) | 0.67 (0.32, 1.42) | 0.35 (0.27, 0.47)* | 1.35 (0.52, 3.63) | 1.69 (0.63, 5.13) | Tofacitinib |  |
| 0.41 (0.15, 1.26) | 0.32 (0.21, 0.48)* | 0.29 (0.13, 0.7)* | 0.24 (0.11, 0.51)* | 0.13 (0.09, 0.17)* | 0.48 (0.17, 1.41) | 0.6 (0.22, 1.84) | 0.36 (0.24, 0.54)* | Upadacitinib |

*means P<0.05

Table S12 League table for endoscopic remission in the ulcerative colitis subgroup

| OR 95%CI | | |
| --- | --- | --- |
| Filgotinib |  |  |
| 2.46 (0.75, 11.54) | Placebo |  |
| 0.39 (0.11, 1.95) | 0.16 (0.09, 0.26)* | Upadacitinib |

*means P<0.05

Table S13 League table for endoscopic improvement in the ulcerative colitis subgroup

| OR 95%CI | | | | | | | |
| --- | --- | --- | --- | --- | --- | --- | --- |
| Brepocitinib |  |  |  |  |  |  |  |
| 6.86 (1.14, 165.5)* | Filgotinib |  |  |  |  |  |  |
| 4.59 (0.58, 115.29) | 0.66 (0.22, 1.78) | Ivarmacitinib |  |  |  |  |  |
| 5.66 (0.76, 142.35) | 0.82 (0.31, 1.99) | 1.23 (0.34, 4.65) | Peficitinib |  |  |  |  |
| 12.4 (2.16, 290.73)* | 1.81 (1.26, 2.66)* | 2.73 (1.1, 7.88)* | 2.21 (1, 5.45)* | Placebo |  |  |  |
| 0.78 (0.47, 1.29) | 0.11 (0, 0.68)* | 0.17 (0.01, 1.33) | 0.14 (0.01, 1.02) | 0.06 (0, 0.36)* | Ritlecitinib |  |  |
| 5.02 (0.84, 120.05) | 0.73 (0.44, 1.24) | 1.11 (0.42, 3.38) | 0.9 (0.37, 2.37) | 0.41 (0.28, 0.58)* | 6.43 (1.09, 153.67)* | Tofacitinib |  |
| 1.48 (0.24, 35.25) | 0.22 (0.12, 0.38)* | 0.33 (0.12, 1.01) | 0.26 (0.11, 0.71)* | 0.12 (0.08, 0.18)* | 1.89 (0.31, 45.32) | 0.29 (0.17, 0.52)* | Upadacitinib |

*means P<0.05

Table S14 League table for adverse events in the ulcerative colitis subgroup

| OR 95%CI | | | | | | | | |
| --- | --- | --- | --- | --- | --- | --- | --- | --- |
| Brepocitinib |  |  |  |  |  |  |  |  |
| 1.01 (0.41, 2.49) | Filgotinib |  |  |  |  |  |  |  |
| 0.6 (0.19, 1.85) | 0.6 (0.27, 1.28) | Ivarmacitinib |  |  |  |  |  |  |
| 0.54 (0.17, 1.62) | 0.53 (0.25, 1.11) | 0.89 (0.33, 2.45) | Peficitinib |  |  |  |  |  |
| 0.85 (0.35, 2.01) | 0.84 (0.64, 1.09) | 1.41 (0.69, 2.95) | 1.57 (0.79, 3.21) | Placebo |  |  |  |  |
| 0.55 (0.15, 1.99) | 0.55 (0.2, 1.45) | 0.92 (0.28, 3.04) | 1.03 (0.32, 3.35) | 0.65 (0.25, 1.67) | Prednisolone |  |  |  |
| 1.2 (0.76, 1.91) | 1.19 (0.48, 2.94) | 2 (0.65, 6.22) | 2.24 (0.74, 6.87) | 1.42 (0.6, 3.37) | 2.18 (0.61, 7.86) | Ritlecitinib |  |  |
| 0.86 (0.34, 2.11) | 0.85 (0.59, 1.23) | 1.42 (0.67, 3.13) | 1.6 (0.77, 3.4) | 1.01 (0.78, 1.31) | 1.55 (0.63, 3.9) | 0.71 (0.29, 1.75) | Tofacitinib |  |
| 0.7 (0.28, 1.76) | 0.7 (0.48, 1.02) | 1.17 (0.55, 2.58) | 1.31 (0.63, 2.81) | 0.83 (0.64, 1.09) | 1.27 (0.48, 3.46) | 0.59 (0.24, 1.45) | 0.82 (0.57, 1.19) | Upadacitinib |

*means P<0.05

Table S15 League table for serious adverse events in the ulcerative colitis subgroup

| OR 95%CI | | | | | | | | |
| --- | --- | --- | --- | --- | --- | --- | --- | --- |
| Brepocitinib |  |  |  |  |  |  |  |  |
| 1.44 (0.17, 41.58) | Filgotinib |  |  |  |  |  |  |  |
| 0.84 (0.02, 41.03) | 0.58 (0.02, 5.3) | Ivarmacitinib |  |  |  |  |  |  |
| 1.83 (0.1, 66.51) | 1.26 (0.15, 6.97) | 2.18 (0.11, 86.26) | Peficitinib |  |  |  |  |  |
| 1.47 (0.2, 41.11) | 1.02 (0.56, 2) | 1.76 (0.22, 51.94) | 0.82 (0.17, 6.52) | Placebo |  |  |  |  |
| 2.21 (0.03, 242.6) | 1.41 (0.03, 52.77) | 2.63 (0.04, 306.41) | 1.17 (0.02, 66.5) | 1.37 (0.04, 47.93) | Prednisolone |  |  |  |
| 1.06 (0.32, 3.56) | 0.74 (0.03, 6.13) | 1.26 (0.03, 60.38) | 0.58 (0.02, 10.54) | 0.72 (0.03, 5.3) | 0.48 (0, 33.37) | Ritlecitinib |  |  |
| 2.58 (0.32, 73.44) | 1.79 (0.78, 4.22) | 3.09 (0.35, 93.54) | 1.43 (0.27, 12.11) | 1.75 (1, 2.99) | 1.28 (0.04, 47.93) | 2.44 (0.3, 70.37) | Tofacitinib |  |
| 2.71 (0.32, 78.58) | 1.88 (0.75, 4.82) | 3.24 (0.35, 102.03) | 1.5 (0.27, 13.05) | 1.83 (0.93, 3.6) | 1.33 (0.04, 54.58) | 2.57 (0.31, 74.12) | 1.05 (0.44, 2.52) | Upadacitinib |

*means P<0.05

Table S16 League table for adverse events leading to treatment discontinuation in the ulcerative colitis subgroup

| OR 95%CI | | | | | | | |
| --- | --- | --- | --- | --- | --- | --- | --- |
| Brepocitinib |  |  |  |  |  |  |  |
| 1.45 (0.17, 47.58) | Filgotinib |  |  |  |  |  |  |
| 1.53 (0.03, 103.27) | 1.02 (0.03, 14.58) | Ivarmacitinib |  |  |  |  |  |
| 0.93 (0.07, 34.77) | 0.64 (0.12, 2.57) | 0.62 (0.03, 25.51) | Peficitinib |  |  |  |  |
| 1.17 (0.16, 36.71) | 0.81 (0.44, 1.55) | 0.79 (0.06, 26.82) | 1.25 (0.37, 5.96) | Placebo |  |  |  |
| 0.44 (0.13, 1.28) | 0.3 (0.01, 2.21) | 0.28 (0, 15.72) | 0.47 (0.01, 5.36) | 0.38 (0.01, 2.4) | Ritlecitinib |  |  |
| 1.5 (0.18, 48.7) | 1.04 (0.43, 2.49) | 1.01 (0.07, 35.66) | 1.61 (0.41, 8.47) | 1.28 (0.68, 2.31) | 3.4 (0.47, 107.08) | Tofacitinib |  |
| 4.9 (0.57, 164.97) | 3.37 (1.33, 9.05)* | 3.31 (0.23, 118.99) | 5.28 (1.27, 29.21)* | 4.14 (2.06, 8.77)* | 11.12 (1.49, 359.64)* | 3.25 (1.29, 8.66)* | Upadacitinib |

*means P<0.05

Table S17 League table for infections in the ulcerative colitis subgroup

| OR 95%CI | | | | | | | | |
| --- | --- | --- | --- | --- | --- | --- | --- | --- |
| Brepocitinib |  |  |  |  |  |  |  |  |
| 5.6 (0.9, 135.35) | Filgotinib |  |  |  |  |  |  |  |
| 11.94 (1.17, 346.8)* | 2.09 (0.48, 7.88) | Ivarmacitinib |  |  |  |  |  |  |
| 7.47 (0.91, 198.01) | 1.32 (0.42, 3.62) | 0.63 (0.12, 3.46) | Peficitinib |  |  |  |  |  |
| 6.73 (1.14, 161.16)* | 1.2 (0.84, 1.77) | 0.58 (0.16, 2.4) | 0.91 (0.36, 2.68) | Placebo |  |  |  |  |
| 17.54 (0.79, 1438.83) | 2.72 (0.26, 82.25) | 1.35 (0.09, 50.3) | 2.11 (0.17, 71.11) | 2.25 (0.23, 67.02) | Prednisolone |  |  |  |
| 2.18 (1.07, 4.61)* | 0.39 (0.02, 2.53) | 0.18 (0.01, 1.92) | 0.29 (0.01, 2.49) | 0.32 (0.01, 2.01) | 0.13 (0, 2.91) | Ritlecitinib |  |  |
| 4.71 (0.76, 113.03) | 0.84 (0.5, 1.41) | 0.4 (0.11, 1.74) | 0.63 (0.23, 1.96) | 0.7 (0.48, 0.99)* | 0.31 (0.01, 3) | 2.16 (0.33, 52.39) | Tofacitinib |  |
| 4.68 (0.72, 115.86) | 0.83 (0.43, 1.59) | 0.4 (0.1, 1.81) | 0.63 (0.21, 2.07) | 0.69 (0.4, 1.16) | 0.31 (0.01, 3.28) | 2.15 (0.31, 53.69) | 0.99 (0.52, 1.87) | Upadacitinib |

*means P<0.05
